# Implications of molecular characters for the phylogeny of the Microbotryaceae (Basidiomycota: Urediniomycetes)

**DOI:** 10.1186/1471-2148-6-35

**Published:** 2006-04-25

**Authors:** Martin Kemler, Markus Göker, Franz Oberwinkler, Dominik Begerow

**Affiliations:** 1 Lehrstuhl für Spezielle Botanik und Mykologie, Botanisches Institut, Universität Tübingen, Auf der Morgenstelle 1, D-72076 Tübingen, Germany

## Abstract

**Background:**

Anther smuts of the basidiomycetous genus *Microbotryum *on Caryophyllaceae are important model organisms for many biological disciplines. Members of *Microbotryum *are most commonly found parasitizing the anthers of host plants in the family Caryophyllaceae, however they can also be found on the anthers of members of the Dipsacaceae, Lamiaceae, Lentibulariaceae, and Portulacaceae. Additionally, some members of *Microbotryum *can be found infecting other organs of mainly Polygonaceae hosts. Based on ITS nrDNA sequences of members of almost all genera in Microbotryaceae, this study aims to resolve the phylogeny of the anther smuts and their relationship to the other members of the family of plant parasites. A multiple analysis strategy was used to correct for the effects of different equally possible ITS sequence alignments on the phylogenetic outcome, which appears to have been neglected in previous studies.

**Results:**

The genera of Microbotryaceae were not clearly resolved, but alignment-independent moderate bootstrap support was achieved for a clade containing the majority of the *Microbotryum *species. The anther parasites appeared in two different well-supported lineages whose interrelationship remained unresolved. Whereas bootstrap support values for some clades were highly vulnerable to alignment conditions, other clades were more robustly supported. The differences in support between the different alignments were much larger than between the phylogenetic optimality criteria applied (maximum parsimony and maximum likelihood).

**Conclusion:**

The study confirmed, based on a larger dataset than previous work, that the anther smuts on Caryophyllaceae are monophyletic and that there exists a native North American group that diverged from the European clade before the radiation of the European species. Also a second group of anther smuts was revealed, containing parasites on Dipsacaceae, Lamiaceae, and Lentibulariaceae. At least the majority of the parasites of Asteraceae appeared as a monophylum, but delimitations of some species in this group should be reconsidered. Parasitism on Polygonaceae is likely to be the ancestral state for the Microbotryaceae on Eudicot hosts.

## Background

### The genera of Microbotryaceae

Anther smuts of the genus *Microbotryum *that parasitise members of the Caryophyllaceae are well-established model organisms. They have been subject to research in different areas, i.e. genetics (e.g. [1]), population analysis (e.g. [2]), phylogenetics (e.g. [3]), host-parasite evolution [4], and ecology [5]. The sorus formation of smuts in the anthers of the hosts is an interesting constellation that has been discussed in the context of pollination. Lateral transmission of the parasite by the pollinator is thought to have a significant effect on the evolutionary history of the genus and its distribution on different hosts [5]. There are ten recognized species of caryophyllaceous anther smuts [6,7], but the species concept in this group is discussed quite controversially. Some authors define at least some species as formae speciales of *Microbotryum violaceum *(Pers.) G. Deml & Oberw. [3,8-10]. In contrast, Liro [11], based on infection experiments and field observations, already separated *Ustilago violacea *(Pers.) Roussel (i.e. *Microbotryum violaceum*) into several species. Molecular studies also indicate genetical isolation of lineages parasitising different host plants [7,10] or occurring in different geographical regions [3]. Against this background, it is desirable to further our understanding of this group. Furthermore, it is noteworthy that the caryophyllaceous anther smuts are only a minor group in *Microbotryum*.

On the basis of ultrastructural features, the Microbotryaceae are defined as phytoparasitic Basidiomycota that have transversely septate basidia with multiple production of sessile basidiospores and intercellular hyphae but no haustoria [12]. The Microbotryaceae are separated from their sister family, the Ustilentylomataceae, by having hyphae with poreless septa at maturity [12]. For an extensive historical overview of *Microbotryum *see [6]. Vánky [6] revised *Microbotryum*, and, based on spore mass colour, transferred most of the *Ustilago *species that parasitise eudicotyledonous plants to *Microbotryum*. Later, new species were added [7,13,14], and *Microbotryum *now contains 77 species. Even though the caryophyllaceous anther smuts are the best-known members of the genus, most species are parasites on Polygonaceae. Hosts are also described in the Asteraceae, Dipsacaceae, Gentianaceae, Lamiaceae, Lentibulariaceae, and Onagraceae. The formation of sori is not restricted to anthers, but there is sorus formation in seeds, whole flowers, pedicels, stems, and leaves.

Next to *Microbotryum *the Microbotryaceae contain *Bauerago *Vánky, *Liroa *Cif., *Sphacelotheca *de Bary, and *Zundeliomyces *Vánky [15]. So far, no hypotheses have been formulated about how these genera might be related to each other. *Sphacelotheca *is distinct from *Microbotryum *by forming appendices between spores, the so-called disjunctors, and by the presence of a columella and a peridium in the sori [16,17]. *Liroa *forms tumours on its host plants including an apical lunular bed of spore masses [16-19]. *Bauerago *is characterized by its parasitism on Cyperaceae and Juncaceae, the presence of a peridium and the lack of a columella [20].

In order to obtain hypotheses about the phylogenetic relationships of the main groups in the Microbotryaceae, we performed molecular phylogenetic analyses based on nuclear internal transcribed spacer (ITS) sequences. Besides *Microbotryum*, specimens of *Bauerago*, *Liroa*, and *Sphacelotheca *were included in our analyses. Thus, with the exception of the monotypic genus *Zundeliomyces*, specimens of which were unavailable to us, all genera in Microbotryaceae were considered. Representative members of Ustilentylomataceae were included for rooting the phylogenetic trees [15].

### Internal transcribed spacer sequences as a molecular marker for smut fungi

The ITS region of the nuclear rDNA has already been used in many studies to resolve phylogenetic relationships within the fungal kingdom. For smut fungi, the ITS has been proven to sufficiently resolve genera, e.g., *Entyloma *[21], *Tilletia *[22], and *Ustilago*/*Sporisorium *[23]. ITS sequences have been used to infer phylogenies of *Microbotryum *and *Sphacelotheca *[7,24], and Freeman et al. [3] demonstrated that the topologies of phylogenetic trees of *M. violaceum *s.l. inferred from ITS data showed no strongly supported inconsistencies to trees inferred from β- and γ-Tubulin. The partition homogeneity test [25] conducted by these authors indicated significant conflict between the partitions, but they discussed the possibility that the partition homogeneity test could be too conservative. Furthermore, the partition homogeneity test has been criticised in general (see [26] and references therein). Based on these studies, it seemed promising to use ITS data to try to answer phylogenetic questions inside the Microbotryaceae.

However, aligning non-coding sequences like ITS may be much more difficult than using protein-coding DNA fragments which are structured by reading frames and have most variability concentrated at third base positions within codons [27]. As Morrison and Ellis [28] have demonstrated, the effects of different underlying DNA sequence alignments on phylogenetic tree reconstruction may be even greater than the effect of the different tree-building methods (e.g., maximum parsimony, maximum likelihood, and distance methods). Tree topologies and branch support inferred from these alignments may be influenced by guide tree topology [29] or input order of sequences [27,30] as well as parameters like the ratios of gap costs to transition/transversions costs [31] used for aligning.

To cope with these problems, one possibility is to exclude the most ambiguously aligned characters before conducting phylogenetic analysis. Gatesy et al. [32] pointed to the advantage of doing this in a reproducible manner. These authors advocated running an alignment program under several parameter combinations and to use only those positions that were consistently revealed under all combinations tested. Excluding alignment-ambiguous regions, however, does not take into account that different possible alignment solutions do not necessarily imply different topologies [33] or support values. An approach based on the generation of a number of alignments by the same algorithm but under different parameter combinations was called "multiple analysis method" by Lee [33]. Here, trees are inferred separately from the respective alignments and only relationships appearing in all (or most) of the trees are accepted (see also [34]). Another possibility would be to use different alignment algorithms under default values, respectively, as did Morrison and Ellis [28]. In addition to the exclusion of ambiguous regions in a reproducible manner we also followed the latter approach and computed trees from three largely different alignments of the same dataset without excluding ambiguous positions. Combining these approaches should reveal whether clade support is based on alignment-ambiguous regions, and, if so, whether it is dependent on how these regions are aligned.

## Results

### DNA alignments

The alignment obtained with MAFFT had a total length of 811 bp. After the exclusion of positions with too many leading or trailing gaps, 738 bp remained, 411 of which were variable and 286 of which were parsimony-informative. The corresponding numbers were 817, 744, 381, and 293 for the PCMA alignment and 850, 749, 370 and 287 for the POA alignment respectively. Considerable parts of the ITS were not identically aligned between these three approaches, including a very long alignment-ambiguous part of the ITS1 and two shorter alignment-ambiguous parts of the ITS2. These alignment ambiguities are also illustrated by the DIALIGN alignment in which 406 of a total of 910 columns had a quality score of only 0 or 1 and were excluded from further analyses. From the remaining 504 positions, 181 were variable and 124 were parsimony-informative.

### Maximum parsimony

**Figure 1:**
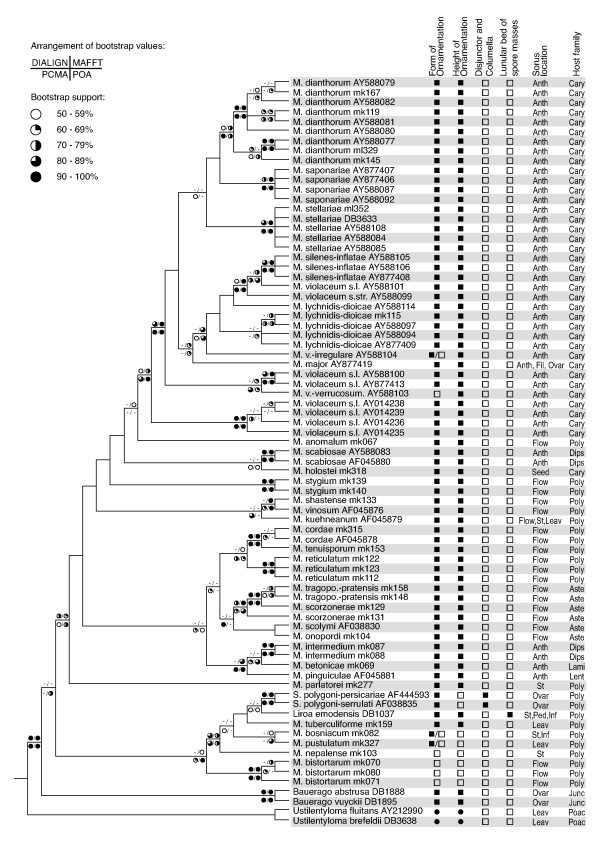
Strict consensus of 1780 most parsimonious trees inferred from the dataset consisting of three concatenated, complete ITS alignments. The topology was rooted with *Ustilentyloma brefeldii *and *U. fluitans*. Symbols on branches indicate the magnitude of parsimony bootstrap values from analyses of the dataset after exclusion of alignment-ambiguous sites (upper left) and of the three different, complete alignments made with MAFFT (upper right), PCMA (lower left), and POA (lower right). The right side of the picture reports morphological features of the specimens included in our data set. The symbols and abbreviations used are as follows. Form of spore ornamentation: filled-in circle, smooth spores; hollow square, verrucose spores; filled-in square, reticulate spores. Height of spore ornamentation: filled-in circle, smooth spores; hollow square, flat ornamentation; filled-in square, high ornamentation. Disjunctors, columella: hollow square, absent; filled-in square, present. Forming galls with an apical lunular bed of spore masses: hollow square, absent; filled-in square, present. Sorus location: Leav, leaves; St, Stems; Inf, inflorescence axis; Ped, pedicels; Flow, swollen (and often deformed) whole flowers; Ovar, ovaries only; Seed, seeds only; Fil, filaments only; Anth, anthers only. Host family: Poac, Poaceae; Junc, Juncaceae; Poly, Polygonaceae; Lent, Lentibulariaceae; Lami, Lamiaceae; Dips, Dipsacaceae; Aste, Asteraceae; Cary, Caryophyllaceae; M., *Microbotryum*; M. tragopo.-pratensis, *Microbotryum tragopogonis-pratensis*; M. v.-irregulare, *Microbotryum violaceo-irregulare*; M. v.-verrucosum, *Microbotryum violaceo-verrucosum*; S., *Sphacelotheca*.

Heuristic maximum parsimony analysis of the concatenated dataset yielded 1780 most parsimonious trees of length 3665. The consistency index of these was 0.513 (0.4963 when uninformative characters were excluded) and the retention index [35] 0.813. Minimum length trees were found in 73 of the 200 replicates. The strict consensus of these most parsimonious trees is shown in Fig. 1 together with bootstrap values obtained by three separate parsimony bootstrap analyses of the MAFFT, PCMA, and POA alignments, respectively. Additionally, maximum parsimony bootstrap values from the reduced dataset obtained by excluding alignment-ambiguous regions are indicated.

**Figure 2:**
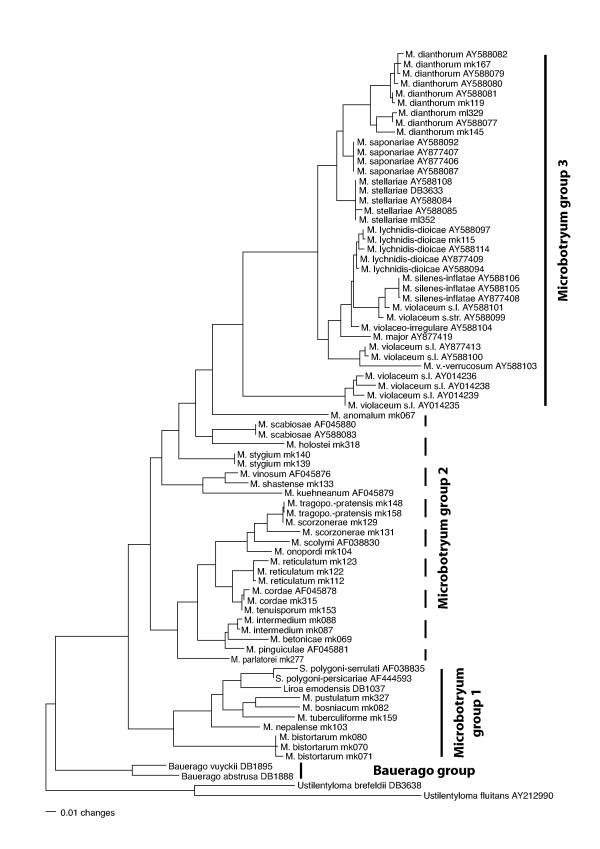
Maximum likelihood analyses of phylogenetic relationships of sampled *Microbotryum *specimens. The tree shown was inferred from the PCMA alignment with PhyML under a TrN+I+G model of site substitution. Branch lengths are scaled in terms of expected numbers of nucleotide substitutions per site. For explanation of the three Microbotryum groups on the right-hand side see discussion. Abbreviations are as in Fig. 1. The dashed line indicates that *Microbotryum *group 2 is not a monophyletic group.

For discussion purposes, we separated the taxa into four distinct informal groups (*Bauerago *group and *Microbotryum *groups I, II, and III; compare Fig. 2). Three of these groups appeared as monophyletic in the analyses, although only two of them were moderately to highly supported as a natural grouping. Group II did not appear as a monophylum, but formed a paraphyletic clade.

Within the ingroup, the two sampled *Bauerago *species, *B. abstrusa *on *Juncus *sp. and *B. vuyckii *on *Luzula *sp. separated basally in the strict consensus, forming a monophyletic group ("*Bauerago *group") with bootstrap values of 99–100% in all alignments. The position of this clade in the tree, however, received bootstrap support only in the POA alignment (79%). If alignment-ambiguous regions were excluded, there was 61% support for an alternative arrangement (not shown).

The *Microbotryum *I group contained *M. bistortarum*, *M. bosniacum*, *M. nepalense*, *M. pustulatum*, and *M. tuberculiforme *as well as the sampled *Sphacelotheca *specimens and the monotypic genus *Liroa*. Support for the group seemed to be especially vulnerable to alignment conditions, ranging from 57% (MAFFT) to 91% (POA). *Microbotryum *group I was unsupported if alignment-ambiguous regions were excluded. There was again 61% support for an arrangement indicating that *M. bistortarum *does not belong to group I (not shown). In the strict consensus inferred from the three concatenated alignments, *M. bistortarum *separated basally within *Microbotryum *group I, its different specimens forming a monophyletic group with support values of 100%.

The following two groups, the apparently paraphyletic *Microbotryum *group II and the apparently monophyletic *Microbotryum *group III, clustered together. This sister-group relationship was weakly to moderately (59–73%) supported under all alignment conditions. Likewise, it received 71% bootstrap support after exclusion of low-quality alignment columns.

*Microbotryum *group II appeared as paraphyletic, although without support, and contained parasites of a broad range of host families including Polygonaceae, Asteraceae, Dipsacaceae, Lentibulariaceae, and Lamiaceae. *M. intermedium *on *Scabiosa *formed a monophyletic group, unsupported if alignment-ambiguous columns were excluded but supported by bootstrap values ranging from 88% (MAFFT) to 99% (POA), otherwise, together with *M. pinguiculae *and *M. betonicae*, parasites in the flowers of Lentibulariaceae and Lamiaceae, respectively. The parasites of members of Asteraceae, *M. tragoponis-pratensis, M. scorzonerae, M. onopordi, and M. scolymi *formed a monophyletic group in strict consensus. However, strong (97%) support for this clade was only revealed with the PCMA alignment. The clade was unsupported under the other alignment conditions and also if alignment-ambiguous regions were excluded. There was, however, strong (97–100%) support under all alignment conditions for the hypothesis that *M. scorzonerae *on *Scorzonera hispanica *is more closely related to *M. tragopogonis-pratensis *than to *M. scorzonerae *on *Scorzonera humilis*.

*M. anomalum*, a smut parasitising the flowers of *Fallopia aubertii *(Polygonaceae), was revealed as the sister group of the caryophyllaceous anther smuts (i.e., *Microbotryum *group III) but with only weak support with the MAFFT alignment (57%) and no support in the other analyses. A seed parasite of *Holosteum umbellatum *(Caryophyllaceae), *M. holostei*, was also situated in *Microbotryum *group II. However, it seems not to be directly related to the anther smuts of Caryophyllaceae since it formed a monophyletic lineage with specimens of *M. scabiosae*, anther smuts on the genus *Knautia *(Dipsacaceae). Yet the support for this relationship was low.

*M. stygium*, which parasitises the flowers of *Rumex acetosa *clustered together with the aforementioned three species and with *Microbotryum *group III, but with no support for its placement. The sister-group relationship of *M. shastense *and *M. vinosum *both of which appear in flowers of Polygonaceae was unsupported by alignment-stable regions but received moderate to strong (69–100%) support from analyses of the three complete alignments, respectively.

*Microbotryum *group III represents the anther smuts of Caryophyllaceae, which formed a monophyletic clade. This clade was hardly supported (57%) after exclusion of alignment-ambiguous columns, but received moderate to strong support (79–90%) from analysis of the three complete alignments, respectively. Among the species described to subdivide *M. violaceum *s.l., *M. dianthorum *got moderate to strong support (74–92%) from all three complete alignments. Bootstrap support for *M. stellariae *ranged from 99 to 100%. The recently described *M. saponariae *was also strongly supported by bootstrap values of 97–100%. These support values were lower if alignment-ambiguous regions were excluded. Relationships between these anther-inhabiting species were generally less well resolved.

### Maximum likelihood

**Figure 3:**
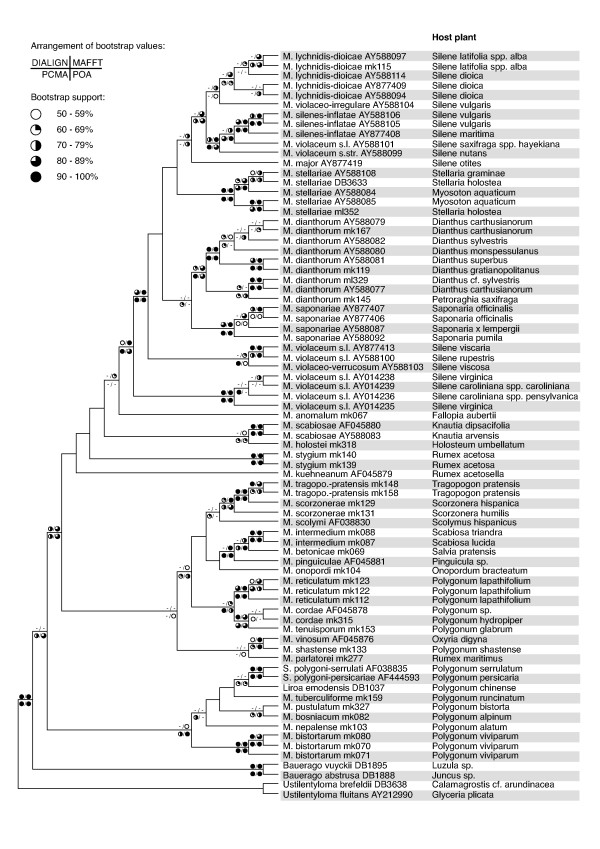
Maximum likelihood majority-rule consensus inferred from the datasets of the three different alignments. The right side of the picture indicates the host plant for each parasite. Symbols on branches indicate the magnitude of likelihood bootstrap values from analyses of the dataset after exclusion of alignment-ambiguous sites (upper left) and of the three different, complete alignments made with MAFFT (upper right), PCMA (lower left), and POA (lower right). Abbreviations are as in Fig. 1.

The substitution models selected by the AICc were TrN+I+G for each of the three complete alignments, respectively, and GTR+I+G for the DIALIGN alignment after exclusion of low quality columns. The tree inferred with PhyML under the best model from the PCMA alignment is shown in Fig. 2. The majority-rule consensus including compatible groupings of the likelihood trees obtained from each of the three complete alignments, respectively, is shown in Fig. 3 together with bootstrap values obtained by separate likelihood bootstrap analyses of the alignments. Additionally, likelihood bootstrap values from the reduced dataset obtained by excluding alignment-ambiguous regions are indicated. In general, support values from likelihood analyses were very similar to the bootstrap results obtained under maximum parsimony and will not be discussed in detail here. The differences in support between the different alignments were much larger than between likelihood and parsimony.

## Discussion

### Methodical aspects

Our multiple analysis approach shows that alignment ambiguities can have a significant impact on branch support obtained by analysis of ITS sequences in *Microbotryum*, a factor which, as in many molecular phylogenetic analyses, has not been investigated so far. Multiple analysis also shows that support for some clades is much more vulnerable to alignment conditions than for other clades. Parsimony bootstrap values indicating monophyly of *Microbotryum *group I, for instance, range from 57% under the MAFFT alignment to 91% under the POA alignment, and the group receives no support from alignment-unambiguous regions alone. *Microbotryum *group III is unsupported (52% parsimony bootstrap) by alignment-stable columns, too, but is considerably supported (79–90% parsimony bootstrap) by analyses of the three complete alignments, respectively. The latter case illustrates the utility of the strategies of Morrison and Ellis [28] and Lee [33] since excluding all the ambiguous positions from the analyses would have resulted in an unnecessary loss of resolution.

Alignments are always just hypotheses of homology of single nucleotides. Alignment positions may be impossible to homologise unambiguously, but if several hypotheses about their homology result in the same groupings in a phylogenetic tree, these groups can be considered supported independently of a specific alignment applied. Furthermore, using automated approaches to alignment avoids the problems of the investigator's bias and the lack of reproducibility that may be related to manual alignments [32].

Partly due to computation time limitations, we only tested three different alignment implementations and probably were unable to fully explore the space of possible solutions to ITS multiple sequence alignment. On the other hand, the three programs we applied are well ranked in simulation studies (e.g., better than the often used CLUSTAL software; [36-38]) and are able to align larger numbers of sequences in reasonable time (in contrast to, e.g., T-Coffee), justifying our selection. Based on this background, we believe the multiple analysis strategy applied here to produce reliable information. The large number of positions not identically aligned between the three alignments made us confident that the alignment space investigated was not too narrow. The influence of different alignments was analysed for the first time in Microbotryaceae and future research should account for these effects, as support values may be quite vulnerable to alignment conditions. The apparently alignment-independent support for other groupings, however, indicates the value of ITS rDNA for phylogenetic purposes in the Microbotryaceae. Furthermore, there is currently no evidence that phylogenetic trees inferred from ITS are more sensitive to alignment conditions in Microbotryaceae than in other groups of comparable rank, because these effects have rarely been investigated.

### Phylogenetics of Microbotryaceae

By means of ITS data we were able to obtain hypotheses on the inter- and intra-generic relationships of the Microbotryaceae (Figs. 1, 2, 3). In the following, the phylogenetic hypotheses obtained will be discussed with a focus on characters, such as morphology and hosts, that are considered to be important in the systematics of the Microbotryaceae [6,12,16,17,39]. The distribution of major traits is, as an overview, mapped on the phylogeny in Fig. 1. With respect to the structure of the spore surface, we found it appropriate to distinguish between the height of the ornamentation and its shape, as we observed four combinations: flat warts, flat meshes, high warts, and high meshes. The other characters are well known from literature.

### Family delimitations

Based on the classification of genera as members of the Microbotryaceae by the absence of a septal porus in mature hyphae [12] we included two *Ustilentyloma *species in the analysis for rooting purposes. Their separation from members of the Microbotryaceae is supported by high bootstrap values under all alignment conditions (Figs. 1, 3). However, more genera of Ustilentylomataceae as well as further outgroup taxa would have to be included in the analysis to support the family concept in the Microbotryales presented by Weiss et al. [15], which is based on ultrastructural characters.

### Bauerago

The lineage in the Microbotryaceae diverging basally is formed by the two *Bauerago *species. This arrangement, however, receives considerable bootstrap support only from the POA alignment. Even though they do not parasitise Poaceae as *Ustilentyloma *does, *Bauerago *species are also parasites of a monocotyledonous family, the Juncaceae. In contrast to *Ustilentyloma*, which forms its sori in leaves, *Bauerago *forms its sori in the ovaries of its host. As the genus *Aurantiosporium*, also a member of the Ustilentylomataceae, forms its sori in the spikelets of Cyperaceae [40], future work is needed to resolve the question if sorus formation in the inflorescence is a plesiomorphic character for the Microbotryaceae.

For the three following groups the phylogenies show that species pathogenic of Polygonaceae are paraphyletic (Figs. 1, 2, 3). Therefore, it seems reasonable to conclude that parasitism on Polygonaceae is the ancestral state for Microbotryaceae on Eudicot hosts.

### *Microbotryum *group I

This group, strongly supported only from the POA alignment and weakly supported under other conditions, manifests a generic clutter, as it contains *Sphacelotheca*, *Liroa*, and *Microbotryum *species (Figs. 1, 3). The group is very diverse in spore ornamentation, height of respective spore ornaments (i.e., warts in case of verrucose spores and meshes of a net-like structure in case of reticulate spores), and location of sorus formation. In general, average height of the spore ornament in this group seems to be much lower than in the other groups [17,39], but as the ornament heights of *Liroa emodensis *and *M. tuberculiforme *show, there are also exceptions to this rule [17,18]. Both species occur on hosts in East Asia [17], but do not appear to be monophyletic. Additionally, spores of many members of this group show a verrucose ornamentation. Only three species of caryophyllaceous anther smuts, *M. chloranthae-verrucosum, M. violaceo-irregulare *and *M. violaceo-verrucosum*, evolved this trait as well, but their spore ornaments seem rather high [7,39]. In addition to verrucose ornamentation there is also reticulate ornamentation in *Microbotryum *group I as in *Liroa emodensis *or *M. tuberculiforme *and, strikingly, in-between forms as in *M. bosniacum *[17,18,39]. Location of sorus formation reaches from the ovaries (e.g. *Sphacelotheca polygoni-serrulati*), the inflorescence axis and the pedicels (*Liroa emodensis*), over the stem (e.g. *M. nepalense*) to the leaves (*M. pustulatum*) and, therefore, must not be considered as being fixed in that group. The only unifying non-molecular character known for this group so far is the parasitism on members of the genus *Polygonum*, a feature, however, that also occurs in members of *Microbotryum *group II. Hence, there is so far no morphological or ecological support for the monophyly of group I. As its bootstrap support values are also highly vulnerable to alignment conditions, the monophyletic status of the group remains doubtful.

The specimens of *M. bistortarum *on *Polygonum viviparum *form a monophyletic lineage that is very well supported by a parsimony and likelihood bootstrap of 100% respectively. As seen in the phylogenetic tree from likelihood analyses (Fig. 2) this group is also separated from the other members of the *Microbotryum *group I by a large genetic distance. Since specimen mk071 has been collected in Mongolia and mk070 and mk080 are from Europe (see Table 1), this lineage exhibits a high uniformity in the ITS sequence even over large geographic distances. Whether or not this is related to the mostly apomictic proliferation of *P. viviparum *[41] remains to be clarified.

**Table 1:** Studied specimens. List of sequenced specimens with hosts, DNA isolation numbers, GenBank  accession numbers, and reference materials. Acronyms: B, Museum Botanicum Berolinense, Berlin, Germany; FO, Franz Oberwinkler, Tübingen, Germany; HUV, Herbarium Ustilaginales Vánky, Tübingen, Germany; M, Botanische Staatssammlung München, Munich, Germany; MP, Meike Piepenbring, Frankfurt, Germany; TUB, Herbarium of the Spezielle Botanik/Mykologie, Eberhard-Karls-Universität Tübingen, Tübingen, Germany.

Species	Host	DNA isolation no.	GenBank accession no.	Reference material
*Bauerago abstrusa *(Malençon) Vánky	*Juncus *sp.	DB1888	DQ238719	HUV 18526
*B. vuyckii *(Oudem. & Beij.) Vánky	*Luzula *sp.	DB1895	DQ238720	MP2380
*Liroa emodensis *(Berk.) Cif.	*Polygonum chinense *L.	DB1037	DQ238743	FO17516
*Microbotryum anomalum *(J. Kunze ex G. Winter) Vánky	*Fallopia aubertii *(L. Henry) Holub	mk067	DQ238721	Hungary, Budapest, Gellért-hegy; leg. K. Imre; 26.10.1983; M-0066114
*M. betonicae *(Beck) R. Bauer & Oberw.	*Salvia pratensis *L.	mk069	DQ238725	Germany, Baden-Württemberg, Tübingen, Spitzberg; leg. A. Nagler, B. Peters, U. & K. Vánky; 19.06.1987; M-0066111
*M. bistortarum *(DC.) Vánky	*Polygonum viviparum *L.	mk080	DQ238711	Germany, Bavaria, Berchtesgaden, Watzmann; leg. H. Schmid-Hechel; 14.07.1982; M-0066099
*M. bistortarum *(DC.) Vánky	*Polygonum viviparum *L.	mk071	DQ238710	Mongolia, Central-Aimak, Chentej; leg. U. Braun; 29.06.1988; M-0066102
*M. bistortarum *(DC.) Vánky	*Polygonum viviparum *L.	mk070	DQ238709	Italy, Friaul, Sauris, Monte Tiarfin; leg. J. Hafellner; U. Trinkaus; 26.07.1995; M-0066101
*M. bosniacum *(G. Beck) Vánky	*Polygonum alpinum *All.	mk082	DQ238740	Italy, Novara, Gries-Pass; leg. F. Oberwinkler, A. Nagler, E., U. & K. Vánky; 13.08.1987; M-0066097
*M. cordae *(Liro) G. Deml & Prillinger	*Polygonum hydropiper *L.	mk315	DQ238726	Germany, Saxony-Anhalt, Kremnitz, Schwarze Elster; leg. H. & I. Scholz; 31.05.2003; B70 0006023
*M. dianthorum *(Liro) H. & I. Scholz	*Dianthus carthusianorum *L.	mk167	DQ238716	Germany, Baden-Württemberg, Tübingen, Unterjesingen; leg. M. Kemler; 31.08.2003; TUB 012503
*M. dianthorum *(Liro) H. & I. Scholz	*Petroraghia saxifraga *(L.) LK	mk145	DQ238718	Italy, Elba, Way E Fetovia 1; leg. M. Hendrichs; 15.05.2000; TUB012106
*M. dianthorum *(Liro) H. & I. Scholz	*Dianthus sylvestris *Wulfen	ml329	DQ238717	Slovenia, Bovec, Trenta, Alpinum Julianum; leg. D. Begerow & M. Lutz; 07.08.2001; TUB012504
*M. dianthorum *(Liro) H. & I. Scholz	*Dianthus gratianopolitanus *Vill.	mk119	DQ238715	Germany, Baden-Württemberg, Tübingen, Bot. Garden; leg. M. Kemler, 04.06.2003; TUB012505
*M. holostei *(de Bary) Vánky	*Holosteum umbellatum *L.	mk318	DQ238722	Germany, Saxony, Sobrigau; leg. M. Siegel; 20.04.2001; B 70 0006032
*M. intermedium *(J. Schröt.) Vánky	*Scabiosa triandra *L.	mk088	DQ238724	Croatia, Krk, Njivice; leg. H. Scholz; 02.08.1979; M-0066091
*M. intermedium *(J. Schröt.) Vánky	*Scabiosa lucida *Vill.	mk087	DQ238723	Germany, Bavaria, Oberjoch, Jochschrofen; leg. K. Vánky; 14.09.1987; M-0066090
*M. lychnidis-dioicae *(DC. ex Liro) G. Deml & Oberw.	*Silene latifolia Poir. ssp. alba *(Mill.) Greuter & Burdet	mk115	DQ238712	Germany, Berlin, Wahlheide; leg. M. Mennicken; 13.06.2003; TUB012506
*M. nepalense *(Liro) Vánky	*Polygonum alatum *Buch.-Ham. Ex D. Don	mk103	DQ238742	India, Uttar Pradesh, Mussoorie, Mt. Gun Hill; leg. K. Vánky; 20.09.1992; M-0066076
*M. onopordi *(Vánky) Vánky	*Onopordum bracteatum *Boiss. & Heldr.	mk104	DQ238735	Greece, Div. Thessalia, Prov Lárisa, pr. Halkiades; leg. D.T. Briese & A. Shepard; 05.07.1989; M-0066075
*M. parlatorei *(A. A. Fisch. Waldh.) Vánky	*Rumex maritimus *L.	mk277	DQ238736	Germany, Saxony-Anhalt, Bleddin, Bleddiner Riβ; leg. I. Scholz; 30.09.2000; B 70 0007574
*M. pustulatum *(DC.) R. Bauer & Oberw.	*Polygonum bistorta *L.	mk327	DQ238741	Germany, Saxony, Mt. Erzgebirge, Hermansdorfer Wiese; leg. W. Dietrich; 03.06.1988; M-0066071
*M. reticulatum *(Liro) R. Bauer & Oberw.	*Polygonum lapathifolium *L.	mk112	DQ238730	Switzerland, Vaud, Yverdon; leg. F. Oberwinkler, A. Nagler, U. & K. Vánky; 12.08.1987; M-0066067
*M. reticulatum *(Liro) R. Bauer & Oberw.	*Polygonum lapathifolium *L.	mk123	DQ238729	Austria, Styria, Windschuh, Kasten; leg. J. Poelt & H. Pittoni; 22.08.1983; M-0066063
*M. reticulatum *(Liro) R. Bauer & Oberw.	*Polygonum lapathifolium *L.	mk122	DQ238728	Bulgaria, Khaskovo, Filevo; leg. K. Imre, S. Vanev & K. Vánky; 08.07.1983; M-0066064
*M. scorzonerae *(Alb. & Schwein.) G. Deml & Prillinger	*Scorzonera humilis *L.	mk131	DQ238734	Germany, Bavaria, Garmisch-Partenkirchen; leg. C. Menge & K. Vánky; 01.06.1991; M-0066056
*M. scorzonerae *(Alb. & Schwein.) G. Deml & Prillinger	*Scorzonera hispanica *L.	mk129	DQ238731	France, Alpes Maritimes, Grasse; leg. A. Nagler & K. Vánky; 09.06.1987; M-0066054
*M. shastense *(Zundel) Vánky	*Polygonum shastense *Brewer	mk133	DQ238739	USA, California, Siskiyou Co., Mt. Shasta; leg. F. Oberwinkler, M. Berbee, G. Thorn & K. Vánky; 08.08.1988; M-0066053
*M. stellariae *(Sowerby) G. Deml & Oberw.	*Stellaria holostea *L.	DB3633	DQ238714	Germany, Baden-Württemberg, Tübingen, Spitzberg; leg. D. Begerow; 26.05.2001; TUB012507
*M. stellariae *(Sowerby) G. Deml & Oberw.	*Stellaria holostea *L.	ml352	DQ238713	Germany, Baden-Württemberg, Ravensburg, Schomburg; leg. M. Kemler; 07.10.2001; TUB012508
*M. stygium *(Liro) Vánky	*Rumex acetosa *L.	mk140	DQ238738	Germany, Baden-Württemberg, Pfullingen, Castle Lichtenstein; leg. M. Berbee & K. Vánky; 11.06.1988; M-0066048
*M. stygium *(Liro) Vánky	*Rumex acetosa *L.	mk139	DQ238737	Germany, Saxony, Erzgebirge, Crottendorf; leg. W. Dietrich; 06.1987; M-0066047
*M. tenuisporum *(Cif.) Vánky	*Polygonum glabrum *Willd.	mk153	DQ238727	India, Karnataka, Mysore; leg. N. D. Sharma, R. Berndt & K. Vánky; 03.11.1995; M-0066041
*M. tragoponis-pratensis *(Pers.) R. Bauer & Oberw.	*Tragopogon pratensis *L.	mk148	DQ238733	Switzerland, Grisons, Sur, Alp Flix; leg. M. Hendrichs; 27.06.2002; TUB012509
*M. tragoponis-pratensis *(Pers.) R. Bauer & Oberw.	*Tragopogon pratensis *L.	mk158	DQ238732	Germany, Thuringia, Themar; leg. H. Dörfelt; 16.06.1986; M-0066039
*M. tuberculiforme *(Syd. & Syd.) Vánky	*Polygonum runcinatum *Hamilt. ex D. Don	mk159	DQ238744	Taiwan, Nan Tou, Mt. Ho Huan San; leg. R. Berndt; 05.07.1990; M-0066035
*Ustilentyloma brefeldii *(Willi Krieg.) Vánky	*Calamagrostis arundinacea *Roth	DB3638	DQ238745	Germany, Baden-Württemberg, Tübingen, Kirnbachtal; leg. H. Vogelmayer; 26.06.01; TUB012510

### *Microbotryum *group II

In general, members of the following two groups form their sori in the host inflorescence but some of the basal members in *Microbotryum *group II develop their sori in stems (*M. parlatorei*) or leaves (*M. kuehneanum*), both this group and the *Microbotryum *group III exhibit high spore ornaments [17,39], and *Microbotryum *group II is additionally characterized by an abundance of host families.

All analyses based on complete alignments show high support values for the monophyly of the group containing *M. intermedium*, *M. betonicae*, and *M. pinguiculae *(Figs. 1, 3); this is an astonishing aspect: even though all of these species form their sori in the anthers they parasitise on different host families. A possible explanation is that all of the latter belong to Euasterids, with Lentibulariaceae and Lamiaceae being very closely related [42]. Another aspect shown by this work is that there could be at least two independent lineages of anther smuts on Dipsacaceae, both in *Microbotryum *group II (Figs. 1, 2, 3). Polyphyly of Dipsacaceae parasites does not receive support in our analyses, however, the sister-group relationship of *M. intermedium *on *Scabiosa *and parasites of Lentibulariaceae and Lamiaceae is well supported. The ITS data cannot reject the hypothesis of monophyly of a group consisting of all the parasites of Dipsacaceae, Lentibulariaceae and Lamiaceae included in this study. Given the monophyly of this group, the most parsimonious interpretation is the paraphyly of the smut fungi on Dipsacaceae with respect to the parasites of the other two families. *M. intermedium *is characterized by a pale spore mass colour whereas *M. scabiosae *on *Knautia *shows a purplish-brown spore mass colour [6]. If spore mass colour corresponds to monophyletic lineages in the dipsacaceous anther smuts, one would predict that *M. succisae *(for which no molecular data were available) clusters with *M. scabiosae *and that *M. flosculorum *and *M. cephalariae *(which were not included in our sample either) cluster with *M. intermedium*. On the other hand, it has been shown that the host genera *Cephalaria *(*M. cephalariae*)*, Succisa *(*M. flosculorum, M. succisae*), and *Knautia *(*M. flosculorum, M. scabiosae*) are more closely related to each other than the hosts of *M. cephalariae *and *M. flosculorum *to *Scabiosa *[43]. Future work in this group will show if host relationships have had a significant effect on parasite phylogeny and if spore mass colour, although rather variable in the Microbotryaceae, is a valuable character in smaller subclades.

The parasites on Asteraceae, based on the presented molecular phylogeny, form a monophyletic group (Figs. 1, 3), although without support. In traditional taxonomy parasites in this group are distinguished by spore mass colour, spore size and host plant. Most interesting is the relationship between *M. tragopogonis-pratensis *and *M. scorzonerae*, as there seem to be parasites on *Scorzonera *that are more closely related to parasites on *Tragopogon *than to other *Scorzonera *parasites; this relationship is strongly supported under all alignment conditions and may indicate that there is a parasite being able to infect both hosts. However, genetic distances between the parasite of *Scorzonera humilis *and *M. tragopogonis-pratensis *are much lower than between these and the parasite of *Scorzonera hispanica *(Fig. 2). Hence, *M. tragopogonis-pratensis *might be able to parasitise some members of *Scorzonera*, but also a separate lineage of *Scorzonera *parasites might exist; this may be due to a host shift from *Tragopogon pratensis *to *Scorzonera humilis *or vice versa. A relatively recent host shift has been described in *Microbotryum violaceum s.l*. from *Petroraghia saxifraga *to *Gypsophila repens *[44]. Additional research, based on, e.g., infection experiments or genotyping, is needed to clarify whether a host jump recently occurred in *M. tragopogonis-pratensis*, too, or whether or not a relatively low host specificity is an ancient condition in this parasite.

### *Microbotryum *group III

Previous phylogenetic studies of members of *Microbotryum *were, with the exception of [24], restricted to caryophyllaceous anther smuts and used other *Microbotryum *species only as outgroups to root trees. Our results, based on a broader species spectrum, confirm the results of previous studies [3,7,24] that the anther smuts of Caryophyllaceae form a monophyletic group. There are several monophyletic clades observed in this group which can be assigned to different parasite species (Figs. 1, 2, 3) and which are consistent with most traditional approaches to the taxonomy of these anther smuts [6]. Based on infection experiments, the splitting of the former *Microbotryum violaceum *s.l. comprising all anther smuts of Caryophyllaceae in a couple of species with narrow host ranges was already proposed by Liro [11]. The large genetic distances within *Microbotryum violaceum *s.l. compared to the distances between and within other *Microbotryum *species (Fig. 2) are in disagreement with its treatment as a single species (comp. [3,10]). Furthermore, the subgroups of *Microbotryum violaceum *s.l. apparently developed stable narrow host specificities as well as stable morphological differences in at least *M. chloranthae-verrucosum*, *M. violaceo-irregulare *and *M. violaceo-verrucosum*, all three with warts on the spore surface, lacking a reticulum [6]. Aiming at a natural classification of this group, future work in anther smuts of Caryophyllaceae will have to split the assemblage into more narrowly defined species, building on the work of Liro [11] and other authors.

Many clades within *Microbotryum violaceum *s.l. are restricted to hosts that are themselves closely related (e.g. *M. dianthorum*, *M. saponariae*, *M. stellariae*; Fig. 2). Indeed, preliminary analyses showed the dominance of cospeciation events in caryophyllaceous anther smuts and their hosts [4]. The trees presented here confirm that the species on the native North American hosts *Silene caroliniana spp. caroliniana, Silene caroliniana spp. pensylvanica *and *Silene virginica *belong to a sister group of the species from native European hosts [3]. This implies that the two groups diverged before the radiation of the European anther smuts. On the other hand, Lutz et al. [7] demonstrated that parasites on the native North American host *Silene douglasii *cluster together with the European *M. lychnidis-dioicae *lineages. Whether this is due to a recent host shift from an invasive host or whether the parasite is also native to North America remains to be clarified.

Sister taxon of the caryophyllaceous anther smuts is *M. anomalum*, a parasite destroying the ovaries and the filaments of *Fallopia aubertii *(Polygonaceae). The seed parasite *M. holostei *on *Holosteum umbellatum *(Caryophyllaceae) forms a monophyletic lineage with two specimens of the dipsacaceous anther smut *M. scabiosae *on *Knautia *species; this lineage appears as a sister taxon of *M. anomalum *and the anther smuts of Caryophyllaceae. The species presumably most closely related to the caryophyllaceous anther smuts raise several new questions about the evolutionary history of parasitism on Caryophyllaceae and of anther smuts in general. If the cluster comprising *Microbotryum *group III, *M. anomalum*, *M. holostei*, and *M. scabiosae *forms a monophylum, their ancestor already had been an anther smut. Our data do not reject this hypothesis. If future work confirms the position of *M. anomalum *between the two lineages of anther smuts, it can be assumed that they have evolved twice independently; this would also imply that the colonization of Caryophyllaceae occurred twice: once in the anthers and once in the seeds. If the position of *M. anomalum *proves to be wrong and the two lineages appear as sister taxa, the ancestor of both might have been a caryophyllaceous anther smut. Afterwards, parasitism in the seeds of Caryophyllaceae could have evolved, leading to *M. holostei*, and another group of parasites originated from a jump on Dipsacaceae as a host family, leading to *M. scabiosae*. That the anther smuts can more or less easily jump from one host family to another is indicated by the fact that anther parasites on three different host families cluster in one group (*M. betonicae*, *M. pinguiculae*, *M. intermedium*; see above and compare Figs. 1, 2). In the light of these questions, it would also be desirable to determine the phylogenetic position of the other seed parasites of Caryophyllaceae and the ovary parasite *M. morinae *on *Morina longifolia *(Dipsacaceae).

As anther smuts parasitise on different host families, the question occurs why radiation was much more extensive in anther smuts of Caryophyllaceae as on the other host families. Previous work has shown that in Europe and North America 113 species of the 849 Caryophyllaceae are hosts for anther smuts [5], which means that about 13.3% are parasitised. In contrast, only 11 (4.4-3.6%) out of the 250–300 species of Dipsacaceae are known to be parasitised by anther smuts [6,39]. Although sampling bias may play a role, it seems implausible that the observed pattern may completely be explained in that way. Nevertheless, future research should make a point of additional sampling effort in anther smuts on host families other than Caryophyllaceae.

### Genus delimitations

The reinstatement of *Microbotryum *by Deml and Oberwinkler [45] only included anther smuts of Caryophyllaceae. Moore [46] proposed that *Ustilago *species of other dicotyledonous hosts should be put into the genus *Bauhinus*. Another approach, mainly based on spore mass colour, was the inclusion of most of the *Ustilago *species on Eudicot hosts into *Microbotryum *[6].

Our molecular data do neither confirm nor reject the *Bauhinus *concept. Taking into account a consequently phylogenetic approach to taxonomy [47], however, it is also evident that an ecological (i.e., with respect to host taxonomy) justification of the proposed genus *Bauhinus *is simply lacking, irrespective of our molecular results. If parasitising Caryophyllaceae is the apomorphic trait, parasitism on other Eudicot families would be plesiomorphic and cannot be used to support monophyly of *Bauhinus*. If parasitising Caryophyllaceae is plesiomorphic (which is highly improbable regarding the molecular phylogenies as well as the distribution of this trait), the monophyletic status of *Microbotryum *s.str. (excluding *Bauhinus*) could not be justified. Hence, the approach of Vánky [6] seems to be the most appropriate taxonomic treatment of the group so far. However, proposals as, e.g., to include *Liroa *into *Microbotryum *[19] should also be considered. Our data do not reject the monophyly of an extended genus *Microbotryum *including *Liroa*.

Our work has shown that the genus delimitations of *Liroa, Microbotryum*, and *Sphacelotheca *need to be reconsidered. Whereas ITS data should be of great help with respect to the delimitation of species (as discussed in the *Microbotryum *group III section) they still provide insufficient evidence with respect to the phylogenetic relationships between genera of the Microbotryaceae in some parts of the tree mostly due to alignment ambiguities. A phylogenetic hypothesis of Microbotryaceae, based on more species or carefully selected species and incorporation of phenotypic as well as additional molecular markers, is needed. A more exhaustive phylogeny may help to understand but may also raise new questions here and in other fields of anther smut research.

## Conclusion

In this study we have used data derived from nuclear ITS and a multiple analysis approach to sequence alignment to address phylogenetic questions in the parasitic fungal family Microbotryaceae. As our analyses confirm the studies of other authors that branch support values for some clades are highly dependent on the alignment approach used, we conclude that more attention should be given to alignment construction in phylogenetic analyses in general. Other clades are robustly supported throughout the alignment space, indicating that the exclusion of ambiguously-aligned regions can lead to a loss of phylogenetically valuable information. Some evidence is presented that the genus *Microbotryum *is paraphyletic, as some *Microbotryum *species form a group with the genera *Liroa *and *Sphacelotheca*, even though support values for this hypothesis are vulnerable to alignment conditions. As the pathogens on Polygonaceae appear to be paraphyletic, we conclude that parasitism on this family forms the ancestral state and parasitism on other plant families is derived. The data also indicate that sorus formation in the reproductive organs of the host is not the derived state but might have been present already in the ancestor of Microbotryaceae. Based on a larger dataset as in previous studies, we could confirm the monophyly of the caryophyllaceous anther smuts and that there exists a group of North American anther smuts on native hosts that is clearly separated from the European clade. Furthermore, the analyses revealed a second clade of anther smuts, containing parasites on Dipsacaceae, Lamiaceae, and Lentibulariaceae, yet, it remains unresolved how the two groups of anther smuts are related to each other. The parasites on Asteraceae form a monophyletic group, although without support.

## Methods

### Sample sources, nomenclature, and morphological information

The specimens examined in this study are listed in Table 1. The nomenclature follows [6,7,39]. Assignment of specimens to species was based on location of sori, spore surface ornamentation, spore mass colour and host data as described by [7,39]. If specimens could not unequivocally be ascribed, the name "*Microbotryum violaceum *s.l." was used as in Vánky [39]. Morphological characters and character state distributions of specimens were compiled from Vánky [6,16,39] and, except characters only observable with scanning electron microscopy, directly checked on the specimens available to us.

### DNA-extraction, PCR, and sequencing

In order to extract genomic DNA the DNeasy™ Plant Mini Kit (Qiagen, Germany) was used. The ITS region localized between the 18S and 28S rRNA genes was amplified by polymerase chain reaction (PCR) using ITS1f and ITS4 [48] or ITS1 and ITS4 [49], respectively, as primers to obtain an approximately 700 bp long DNA fragment. To purify the PCR products, the QIAquick™ Kit (Qiagen, Germany) was used. Samples were sequenced with the BigDye™ Terminator Cycle Sequencing Kit V3.1 (Applied Biosystems) on an automatic sequencer (ABI 3100 Genetic Analyser). DNA sequences determined in the course of this study were deposited in GenBank, accession numbers are given in Table 1.

Additionally, the following sequences from GenBank were used: [AF045876, AF045878, AF045879, AF045880, AF045881, AY212990, AY014235, AY014236, AY014238, AY014239, AY588077, AY588079, AY588080, AY588081, AY588082, AY588083, AY588084, AY588085, AY588087, AY588092, AY588094, AY588097, AY588099, AY588100, AY588101, AY588103, AY588104, AY588105, AY588106, AY588108, AY588114, AY877406, AY877407, AY877408, AY877409, AY877413, AY877419].

### Sequence alignment

As described above, we followed both a "multiple analysis" [33] as well as an exclusion strategy to explore the alignment-dependency of the phylogenetic results. For exclusion of alignment-ambiguous regions, ITS sequences were aligned with DIALIGN 2.2.1 [50] using the -n option. In contrast to the majority of alignment tools, DIALIGN constructs a multiple sequence alignment (including a quality score for each alignment column) from whole sequence fragments found in local pair-wise alignments. All positions that obtained a quality score as low as 0 or 1 were excluded before inferring trees; this procedure automatically lead to the deletion of positions with a lot of leading or trailing gaps due to incomplete sequencing too. Additionally, DIALIGN frequently regards stretches of bases in single sequences as unaligned; these were coded as missing data.

For multiple analysis, MAFFT [36] was used under the FFT-NS-i option, i.e. with fast construction of an initial alignment followed by iterative refinement until convergence. The POA software [51] uses the partial order graph format to effectively store alignment information. POA was run in progressive and local mode (-do_progressive -read_pairscores <filename>) after computing pair-wise sequence similarities with the make_pscores.pl script provided with the POA package (we slightly modified the script for use with DNA BLAST instead of protein BLAST). PCMA [38] uses the faster Clustal algorithm [52] for similar sequences and the slower but more accurate T-Coffee algorithm [53] for more divergent sequences. We used -ave_grp_id = 90, i.e. forced all sequence groups with less than 90% identity to be aligned with T-Coffee.

Positions of many leading or trailing gaps were excluded before inferring phylogenetic trees. Cross-comparison between the three alignments also revealed that some short (about 2–10 bp) stretches of bases in a few sequences in two of the alignments were likely to be misaligned due to incomplete sequencing resulting in long leading or trailing gaps in these sequences; these stretches were coded as missing data. No manual "corrections" were applied as recommended by Giribet and Wheeler [54] and Gatesy et al. [32].

Maximum parsimony heuristic search (as explained below) was then performed on a data matrix obtained by concatenating these three alignments, similar to the elision method of Wheeler et al. [31]. Bootstrapping, however, requires statistical independency of all alignment positions [33]. Accordingly, bootstrap analyses were performed separately for the different alignments. In principle, a common majority-rule consensus can then be computed to summarize the results of such a "multiple analysis" [33], as discussed in Farris et al. [34]. However, we preferred to show the different support values as in Morrison and Ellis [28], since the magnitude of the difference between the bootstrap supports inferred from different alignments reveals how sensitive the respective clades were to the alignment algorithm applied.

### Phylogenetic analysis

With PAUP*, a heuristic search under the maximum parsimony criterion (e.g., [55]) was performed using 200 replicates with random addition of sequences and subsequent TBR branch swapping (multrees option in effect, steepest descent option not in effect). Gaps were treated as missing data. Due to the large number of equally parsimonious trees found, no more than 25 trees of score greater than or equal to 1 were saved in each replicate. Bootstrap analysis [56] under maximum parsimony was conducted with PAUP* as well using 1000 replicates. In each bootstrap replicate, 25 random sequence addition replicates followed by TBR search were performed, saving no more than 10 trees of score greater than or equal to 1 per replicate and using a reconnection limit of 12. Uninformative characters were excluded before bootstrapping.

To obtain appropriate substitution models for maximum likelihood analysis, each alignment was analysed with Modeltest 3.6 [57] using the Akaike information criterion in its AICc variant [58]. Under the respective model found, likelihood analyses were then performed with PhyML 2.4.4 [59]. For bootstrapping, PhyML's built-in bootstrap function was used with 500 replicates, respectively.

Based on studies of [15], two *Ustilentyloma *species belonging to the Ustilentylomataceae, which is the sister group of the Microbotryaceae, were used to root the trees. All alignments together with the trees inferred from them were deposited in TREEBASE [60] and are included as files in NEXUS format with comments in the supplementary material [see Additional file 1].

## Authors' contributions

MK collected specimens in the field; obtained most of the genomic DNA; conducted the majority of the ITS rDNA sequencing; and wrote the manuscript. MG conducted the sequence alignments; performed the phylogenetic analyses; and wrote the manuscript. MK and MG collected the morphological data. FO provided ideas and support for phylogenetic work. DB collected specimens in the field; isolated some of the genomic DNA; revised the text; introduced MK to the field of molecular systematics; and supervised his Ph.D. thesis. All authors read and approved the final manuscript.

## Supplementary Material

Additional File 1 Alignment, Bootstrap trees, Trees. Dialign NEXUS file and concatenated MAFFT, PCMA, and POA NEXUS file. Likelihood and Parsimony bootstrap .tre and .log files and PhyML bootstrap .rtf file. Likelihood and Parsimony .tre, .log and .con files. Click here for file
