# Supplementary material for: Implications of molecular characters for the phylogeny of the Microbotryaceae (Basidiomycota: Urediniomycetes)
Source: BMC Evol Biol. 2006 Apr 25;6:35. doi: 10.1186/1471-2148-6-35 (PMC1526759; doi:10.1186/1471-2148-6-35)
Supplement: Additional File 1 — Alignment, Bootstrap trees, Trees. Dialign NEXUS file and concatenated MAFFT, PCMA, and POA NEXUS file. Likelihood and Parsimony bootstrap .tre and .log files and PhyML bootstrap .rtf file. Likelihood and Parsimony .tre, .log and .con files. [file 1471-2148-6-35-S1.zip › Bootstrap_Trees/Likelihood/phyml_bootstrap.rtf]

Processing of file "E:\Markus\Microbotryum\Mic_Martin\combined\phyml\mafft_phyml_boot.tre" completed.

Outgroup status changed:
  2 taxa transferred to outgroup
  Total number of taxa now in outgroup = 2
  Number of ingroup taxa = 75

50% Majority-rule consensus of 500 trees

                                                                                         /-------- M. lychnidis dioicae ml326(1)
                                                                                 /--81---+-------- M. lychnidis dioicae mk115(2)
                                                                         /--63---+---------------- M. lychnidis dioicae DB3636(5)
                                                                 /--89---+------------------------ M. lychnidis dioicae ml215(3)
                                                         /--73---+       \------------------------ M. lychnidis dioicae mk147(4)
                                                         |       \-------------------------------- M. violaceo irregulare ml50(11)
                                                         |                               /-------- M. silenes inflatae ml620(9)
                                                /---87---+                       /--99---+-------- M. silenes inflatae ml605(10)
                                                |        |               /--100--+---------------- M. silenes inflatae mk208(8)
                                        /--75---+        |       /--77---+------------------------ M. silenes inflatae ml327(7)
                                        |       |        \--90---+-------------------------------- M. violaceum s.str. ml344(6)
                                        |       \------------------------------------------------- M. major mk279(12)
                                        |                                                /-------- M. dianthorum ml333(22)
                                        |                                                +-------- M. dianthorum ml354(23)
                                        |                                        /--59---+-------- M. dianthorum ml328(24)
                                        |                                        |       \-------- M. dianthorum mk167(25)
                                        |                                /--99---+       /-------- M. dianthorum ml622(26)
                                        |                                |       \--97---+-------- M. dianthorum mk119(27)
                                        |                        /--65---+               /-------- M. dianthorum ml329(28)
                                        +-----------87-----------+       \------100------+-------- M. dianthorum DB3637(29)
                                        |                        \-------------------------------- M. dianthorum mk145(30)
                                /--100--+                                                /-------- M. stellariae ml507(16)
                                |       |                                        /--71---+-------- M. stellariae DB3633(17)
                                |       |                                /--86---+---------------- M. stellariae ml48(15)
                                |       +--------------100---------------+               /-------- M. stellariae HML791(13)
                                |       |                                \------93-------+-------- M. stellariae ml352(14)
                                |       |                                                /-------- M. saponariae mk146(18)
                                |       |                                        /--96---+-------- M. saponariae mk142(19)
                                |       |                                /--86---+---------------- M. saponariae ml310(20)
                        /--90---+       +--------------100---------------+------------------------ M. saponariae ml373(21)
                        |       |       |                                                /-------- M. violaceum s.l. mk143(31)
                        |       |       |                                        /--96---+-------- M. violaceum s.l. mk113(32)
                        |       |       \-------------------63-------------------+---------------- M. violaceo verrucosum ml49(33)
                /--66---+       |                                                        /-------- M. violaceum s.l. AY014239(34)
                |       |       |                                                /--79---+-------- M. violaceum s.l. AY014238(35)
                |       |       |                                        /--73---+---------------- M. violaceum s.l. AY014235(37)
                |       |       \------------------100-------------------+------------------------ M. violaceum s.l. AY014236(36)
                |       \------------------------------------------------------------------------- M. anomalum mk067(38)
                |                                                                        /-------- M. tenuisporum mk153(54)
                |                                                                /--57---+-------- M. cordae mk315(56)
                |                                                        /--87---+---------------- M. cordae AF045878(55)
                |                                                        |               /-------- M. reticulatum mk123(57)
                |                                                /--66---+       /--80---+-------- M. reticulatum mk122(58)
                |                                                |       \--100--+---------------- M. reticulatum mk112(59)
        /--81---+                                                |                       /-------- M. intermedium mk088(45)
        |       |                                                |               /--100--+-------- M. intermedium mk087(46)
        |       |                                                |       /--78---+---------------- M. betonicae mk069(44)
        |       |                                                +--94---+------------------------ M. pinguiculae AF045881(47)
        |       |                                        /--52---+                       /-------- M. tragopo. pratensis mk148(49)
        |       |                                        |       |               /--80---+-------- M. tragopo. pratensis mk158(50)
        |       |                                        |       |       /--99---+---------------- M. scorzonerae mk129(51)
        |       |                                        |       +--97---+------------------------ M. scorzonerae mk131(48)
        |       |                                        |       +-------------------------------- M. scolymi NO1(52)
        |       |                                        |       \-------------------------------- M. onopordi mk104(53)
        |       |                                        |                               /-------- M. scabiosae AF045880(64)
        |       \-------------------58-------------------+                       /--100--+-------- M. scabiosae NO3(65)
        |                                                +----------54-----------+---------------- M. holostei mk318(63)
        |                                                |                               /-------- M. vinosum AF045876(41)
        |                                                +--------------98---------------+-------- M. shastense mk133(42)
/-------+                                                |                               /-------- M. stygium mk140(60)
|       |                                                +--------------100--------------+-------- M. stygium mk139(61)
|       |                                                +---------------------------------------- M. parlatorei mk277(43)
|       |                                                \---------------------------------------- M. kuehneanum AF045879(62)
|       |                                                                                /-------- M. bistortarum mk080(74)
|       |                                                                        /--86---+-------- M. bistortarum mk070(75)
|       |                                                                /--100--+---------------- M. bistortarum mk071(73)
|       |                                                                |               /-------- Sphac. poly. serrulati NO2(67)
|       |                                                                +------99-------+-------- Sphac. poly. persicariae AF444593(68)
100     |                                                                +------------------------ M. tuberculiforme mk159(66)
|       +-------------------------------57-------------------------------+------------------------ M. bosniacum mk082(69)
|       |                                                                +------------------------ M. pustulatum mk327(70)
|       |                                                                +------------------------ Liroa emodensis DB1037(71)
|       |                                                                \------------------------ M. nepalense mk103(72)
|       |                                                                                /-------- Bauerago vuyckii DB1895(39)
|       \--------------------------------------100---------------------------------------+-------- Bauerago abstrusa DB1888(40)
|                                                                                        /-------- Ustilentyloma spec. 3638(76)
\----------------------------------------------------------------------------------------+-------- Ustilentyloma fluitans 1040(77)


 Processing of file "E:\Markus\Microbotryum\Mic_Martin\combined\phyml\pcma_phyml_boot.tre" completed.

Outgroup status changed:
  2 taxa transferred to outgroup
  Total number of taxa now in outgroup = 2
  Number of ingroup taxa = 75

50% Majority-rule consensus of 500 trees

                                                                                         /-------- M. dianthorum mk167(28)
                                                                                         +-------- M. dianthorum ml328(29)
                                                                                 /--61---+-------- M. dianthorum ml354(30)
                                                                                 |       \-------- M. dianthorum ml333(31)
                                                                         /--98---+       /-------- M. dianthorum ml622(32)
                                                                         |       \--96---+-------- M. dianthorum mk119(33)
                                                                 /--97---+               /-------- M. dianthorum ml329(26)
                                                                 |       |       /--100--+-------- M. dianthorum DB3637(27)
                                                                 |       \--62---+---------------- M. dianthorum mk145(25)
                                                         /--62---+                       /-------- M. saponariae mk142(17)
                                                         |       |               /--55---+-------- M. saponariae mk146(18)
                                                         |       |       /--54---+---------------- M. saponariae ml310(19)
                                                         |       \--100--+------------------------ M. saponariae ml373(16)
                                                /---90---+                               /-------- M. stellariae ml507(23)
                                                |        |                       /--78---+-------- M. stellariae DB3633(24)
                                                |        |               /--87---+---------------- M. stellariae ml48(22)
                                                |        \------100------+               /-------- M. stellariae HML791(20)
                                                |                        \------83-------+-------- M. stellariae ml352(21)
                                                |                                        /-------- M. silenes inflatae ml620(2)
                                                |                                /--100--+-------- M. silenes inflatae ml605(3)
                                                |                        /--100--+---------------- M. silenes inflatae mk208(1)
                                                |                /--86---+------------------------ M. silenes inflatae ml327(4)
                                                |        /--97---+-------------------------------- M. violaceum s.str. ml344(5)
                                        /--100--+        |                               /-------- M. lychnidis dioicae ml326(7)
                                        |       |        |                       /--79---+-------- M. lychnidis dioicae mk115(8)
                                        |       +---71---+                       +---------------- M. lychnidis dioicae DB3636(6)
                                        |       |        +----------62-----------+---------------- M. lychnidis dioicae ml215(9)
                                        |       |        |                       \---------------- M. lychnidis dioicae mk147(10)
                                        |       |        \---------------------------------------- M. violaceo irregulare ml50(11)
                                        |       |                                        /-------- M. violaceum s.l. mk143(13)
                        /------92-------+       |                                /--77---+-------- M. violaceum s.l. mk113(14)
                        |               |       +---------------90---------------+---------------- M. violaceo verrucosum ml49(15)
                        |               |       \------------------------------------------------- M. major mk279(12)
                        |               |                                                /-------- M. violaceum s.l. AY014236(36)
                        |               |                                        /--82---+-------- M. violaceum s.l. AY014238(37)
                        |               |                                /--92---+---------------- M. violaceum s.l. AY014239(34)
                        |               \--------------100---------------+------------------------ M. violaceum s.l. AY014235(35)
                        |                                                                /-------- M. tragopo. pratensis mk148(54)
                        |                                                        /--63---+-------- M. tragopo. pratensis mk158(55)
                        |                                                /--99---+---------------- M. scorzonerae mk129(56)
                        |                                        /--73---+------------------------ M. scorzonerae mk131(53)
                        |                                /--68---+-------------------------------- M. scolymi NO1(52)
                        |                       /---99---+---------------------------------------- M. onopordi mk104(51)
                        |                       |                                        /-------- M. cordae AF045878(58)
                        |                       |                                /--59---+-------- M. cordae mk315(59)
                        |               /--72---+                        /--85---+---------------- M. tenuisporum mk153(57)
                /--71---+               |       |                        |               /-------- M. reticulatum mk123(61)
                |       |               |       \-----------90-----------+       /--81---+-------- M. reticulatum mk122(62)
                |       |               |                                \--100--+---------------- M. reticulatum mk112(60)
                |       |       /--60---+                                                /-------- M. intermedium mk088(49)
                |       |       |       |                                        /--99---+-------- M. intermedium mk087(50)
                |       +--86---+       |                                /--78---+---------------- M. betonicae mk069(48)
                |       |       |       \---------------98---------------+------------------------ M. pinguiculae AF045881(47)
                |       |       \----------------------------------------------------------------- M. parlatorei mk277(63)
                |       |                                                                /-------- M. shastense mk133(38)
                |       |                                                        /--69---+-------- M. vinosum AF045876(39)
                |       +---------------------------97---------------------------+---------------- M. kuehneanum AF045879(40)
                |       |                                                                /-------- M. scabiosae AF045880(43)
        /--75---+       |                                                        /--100--+-------- M. scabiosae NO3(44)
        |       |       +---------------------------62---------------------------+---------------- M. holostei mk318(41)
        |       |       |                                                                /-------- M. stygium mk140(45)
        |       |       +------------------------------100-------------------------------+-------- M. stygium mk139(46)
        |       |       \------------------------------------------------------------------------- M. anomalum mk067(42)
        |       |                                                                        /-------- M. pustulatum mk327(68)
        |       |                                                                /--61---+-------- M. bosniacum mk082(71)
        |       |                                                        /--70---+---------------- M. tuberculiforme mk159(74)
/-------+       |                                                        |               /-------- Sphac. poly. serrulati NO2(69)
|       |       |                                                /--72---+       /--100--+-------- Sphac. poly. persicariae AF444593(70)
|       |       |                                                |       +--65---+---------------- Liroa emodensis DB1037(73)
|       |       |                                                |       \------------------------ M. nepalense mk103(72)
|       |       \-----------------------73-----------------------+                       /-------- M. bistortarum mk080(76)
100     |                                                        |               /--94---+-------- M. bistortarum mk070(77)
|       |                                                        \------100------+---------------- M. bistortarum mk071(75)
|       |                                                                                /-------- Bauerago vuyckii DB1895(66)
|       \--------------------------------------100---------------------------------------+-------- Bauerago abstrusa DB1888(67)
|                                                                                        /-------- Ustilentyloma spec. 3638(64)
\----------------------------------------------------------------------------------------+-------- Ustilentyloma fluitans 1040(65)


Processing of file "E:\Markus\Microbotryum\Mic_Martin\combined\phyml\poa_phyml_boot.tre" completed.

Outgroup status changed:
  2 taxa transferred to outgroup
  Total number of taxa now in outgroup = 2
  Number of ingroup taxa = 75

50% Majority-rule consensus of 500 trees

                                                                                         /-------- M. lychnidis dioicae mk147(13)
                                                                                 /--79---+-------- M. lychnidis dioicae ml215(14)
                                                                         /--62---+       /-------- M. lychnidis dioicae ml326(15)
                                                                 /--69---+       \--80---+-------- M. lychnidis dioicae mk115(16)
                                                         /--50---+       \------------------------ M. lychnidis dioicae DB3636(17)
                                                         |       \-------------------------------- M. violaceo irregulare ml50(12)
                                                         |                               /-------- M. silenes inflatae ml620(10)
                                                /---88---+                       /--99---+-------- M. silenes inflatae ml605(11)
                                                |        |               /--100--+---------------- M. silenes inflatae mk208(9)
                                        /--71---+        |       /--94---+------------------------ M. silenes inflatae ml327(8)
                                        |       |        \--87---+-------------------------------- M. violaceum s.str. ml344(7)
                                        |       \------------------------------------------------- M. major mk279(6)
                                        |                                                /-------- M. dianthorum mk167(28)
                                        |                                        /--60---+-------- M. dianthorum ml354(30)
                                        |                                /--70---+---------------- M. dianthorum ml328(29)
                                        |                                |               /-------- M. dianthorum ml622(26)
                                        |                        /--97---+------93-------+-------- M. dianthorum mk119(27)
                                        |                        |       \------------------------ M. dianthorum ml333(25)
                                        +-----------87-----------+                       /-------- M. dianthorum ml329(23)
                                        |                        |               /--100--+-------- M. dianthorum DB3637(24)
                                        |                        \------78-------+---------------- M. dianthorum mk145(22)
                                /--98---+                                                /-------- M. stellariae ml507(1)
                                |       |                                        /--79---+-------- M. stellariae DB3633(2)
                                |       |                                /--92---+---------------- M. stellariae ml48(3)
                                |       +--------------100---------------+               /-------- M. stellariae HML791(4)
                                |       |                                \------92-------+-------- M. stellariae ml352(5)
                                |       |                                                /-------- M. saponariae mk146(20)
                                |       |                                        /--56---+-------- M. saponariae mk142(21)
                        /--89---+       |                                /--56---+---------------- M. saponariae ml310(19)
                        |       |       +--------------100---------------+------------------------ M. saponariae ml373(18)
                        |       |       |                                                /-------- M. violaceum s.l. mk143(31)
                        |       |       |                                        /--90---+-------- M. violaceum s.l. mk113(32)
                        |       |       \-------------------57-------------------+---------------- M. violaceo verrucosum ml49(33)
                        |       |                                                        /-------- M. violaceum s.l. AY014239(34)
                        |       |                                                        +-------- M. violaceum s.l. AY014235(35)
                        |       \--------------------------100---------------------------+-------- M. violaceum s.l. AY014236(36)
                        |                                                                \-------- M. violaceum s.l. AY014238(37)
                        |                                                                /-------- M. reticulatum mk123(52)
                        |                                                        /--77---+-------- M. reticulatum mk122(53)
                        |                                                /--99---+---------------- M. reticulatum mk112(54)
                        |                                        /--74---+               /-------- M. tenuisporum mk153(55)
                        |                                        |       \------86-------+-------- M. cordae AF045878(56)
                        |                                        |                       \-------- M. cordae mk315(57)
                        |                                        |                       /-------- M. intermedium mk088(45)
                        |                                        |               /--99---+-------- M. intermedium mk087(46)
                /--88---+                                        |       /--98---+---------------- M. betonicae mk069(44)
                |       |                                /--70---+--100--+------------------------ M. pinguiculae AF045881(43)
                |       |                                |       |                       /-------- M. tragopo. pratensis mk148(48)
                |       |                                |       |               /--73---+-------- M. tragopo. pratensis mk158(49)
                |       |                                |       |       /--100--+---------------- M. scorzonerae mk129(50)
                |       |                                |       +--83---+------------------------ M. scorzonerae mk131(51)
                |       +---------------54---------------+       +-------------------------------- M. onopordi mk104(42)
                |       |                                |       \-------------------------------- M. scolymi NO1(47)
                |       |                                |                               /-------- M. vinosum AF045876(58)
                |       |                                |                       /--100--+-------- M. shastense mk133(59)
                |       |                                \----------56-----------+---------------- M. parlatorei mk277(60)
                |       |                                                                /-------- M. scabiosae AF045880(40)
        /--85---+       |                                                        /--100--+-------- M. scabiosae NO3(41)
        |       |       +---------------------------65---------------------------+---------------- M. holostei mk318(39)
        |       |       |                                                                /-------- M. stygium mk140(61)
        |       |       +------------------------------100-------------------------------+-------- M. stygium mk139(62)
        |       |       +------------------------------------------------------------------------- M. anomalum mk067(38)
        |       |       \------------------------------------------------------------------------- M. kuehneanum AF045879(63)
        |       |                                                                        /-------- Sphac. poly. serrulati NO2(70)
        |       |                                                                /--100--+-------- Sphac. poly. persicariae AF444593(71)
        |       |                                                        /--61---+---------------- Liroa emodensis DB1037(69)
/-------+       |                                                        |               /-------- M. bistortarum mk080(76)
|       |       |                                                        |       /--90---+-------- M. bistortarum mk070(77)
|       |       |                                                        +--100--+---------------- M. bistortarum mk071(75)
|       |       \---------------------------92---------------------------+               /-------- M. pustulatum mk327(73)
|       |                                                                +------78-------+-------- M. bosniacum mk082(74)
99      |                                                                +------------------------ M. tuberculiforme mk159(68)
|       |                                                                \------------------------ M. nepalense mk103(72)
|       |                                                                                /-------- Bauerago vuyckii DB1895(66)
|       \---------------------------------------99---------------------------------------+-------- Bauerago abstrusa DB1888(67)
|                                                                                        /-------- Ustilentyloma spec. 3638(64)
\----------------------------------------------------------------------------------------+-------- Ustilentyloma fluitans 1040(65)


Processing of file "E:\Markus\Microbotryum\Mic_Martin\combined\phyml\dialign01_phyml_boot.tre" completed.

Outgroup status changed:
  2 taxa transferred to outgroup
  Total number of taxa now in outgroup = 2
  Number of ingroup taxa = 75

50% Majority-rule consensus of 500 trees

                                                                                     /------------ M. dianthorum ml328(25)
                                                                                     +------------ M. dianthorum ml354(26)
                                                                         /----67-----+------------ M. dianthorum ml333(27)
                                                                         |           \------------ M. dianthorum mk167(28)
                                                             /----97-----+           /------------ M. dianthorum mk119(29)
                                                             |           \----80-----+------------ M. dianthorum ml622(30)
                                                /-----64-----+                       /------------ M. dianthorum ml329(23)
                                                |            |           /----99-----+------------ M. dianthorum DB3637(24)
                                                |            \----60-----+------------------------ M. dianthorum mk145(22)
                                                |                                    /------------ M. stellariae ml507(4)
                                                |                        /----59-----+------------ M. stellariae DB3633(5)
                                                |            /----67-----+------------------------ M. stellariae ml48(6)
                                                +-----96-----+                       /------------ M. stellariae HML791(2)
                                                |            \----------98-----------+------------ M. stellariae ml352(3)
                                                |                                    /------------ M. silenes inflatae ml620(15)
                                                |                        /----71-----+------------ M. silenes inflatae ml605(16)
                                                |            /----89-----+------------------------ M. silenes inflatae mk208(14)
                                                +-----72-----+------------------------------------ M. violaceum s.str. ml344(13)
                                                |            \------------------------------------ M. silenes inflatae ml327(17)
                                    /----84-----+                                    /------------ M. saponariae mk146(18)
                                    |           |                        /----71-----+------------ M. saponariae mk142(19)
                                    |           +-----------85-----------+           /------------ M. saponariae ml310(20)
                                    |           |                        \----90-----+------------ M. saponariae ml373(21)
                                    |           |                                    /------------ M. violaceum s.l. mk143(8)
                                    |           +-----------------92-----------------+------------ M. violaceum s.l. mk113(9)
                                    |           |                                    /------------ M. lychnidis dioicae mk115(10)
                                    |           +-----------------52-----------------+------------ M. lychnidis dioicae mk147(11)
                        /----51-----+           +------------------------------------------------- M. lychnidis dioicae DB3636(1)
                        |           |           +------------------------------------------------- M. major mk279(7)
                        |           |           +------------------------------------------------- M. violaceo irregulare ml50(12)
                        |           |           +------------------------------------------------- M. violaceo verrucosum ml49(36)
                        |           |           +------------------------------------------------- M. lychnidis dioicae ml215(37)
                        |           |           \------------------------------------------------- M. lychnidis dioicae ml326(38)
                        |           |                                                /------------ M. violaceum s.l. AY014235(32)
                        |           |                                    /----57-----+------------ M. violaceum s.l. AY014238(34)
                        |           \----------------100-----------------+------------------------ M. violaceum s.l. AY014236(31)
                        |                                                \------------------------ M. violaceum s.l. AY014239(33)
                        |                                                            /------------ M. tragopo. pratensis mk158(56)
                        |                                                /----100----+------------ M. tragopo. pratensis mk148(57)
                        |                                    /----100----+------------------------ M. scorzonerae mk129(55)
                        +-----------------69-----------------+------------------------------------ M. scorzonerae mk131(54)
                        |                                                            /------------ M. tenuisporum mk153(51)
                        |                                                /----68-----+------------ M. cordae mk315(52)
                        +-----------------------91-----------------------+------------------------ M. cordae AF045878(53)
                        |                                                            /------------ M. reticulatum mk112(63)
            /----74-----+                                                /----53-----+------------ M. reticulatum mk123(64)
            |           +-----------------------86-----------------------+------------------------ M. reticulatum mk122(65)
            |           |                                                            /------------ M. scabiosae AF045880(40)
            |           +----------------------------100-----------------------------+------------ M. scabiosae NO3(41)
            |           |                                                            /------------ M. stygium mk140(42)
            |           +----------------------------100-----------------------------+------------ M. stygium mk139(43)
            |           |                                                            /------------ M. intermedium mk088(48)
            |           +-----------------------------99-----------------------------+------------ M. intermedium mk087(49)
            |           |                                                            /------------ M. kuehneanum AF045879(58)
            |           +-----------------------------52-----------------------------+------------ M. scolymi NO1(62)
            |           |                                                            /------------ M. shastense mk133(59)
            |           +-----------------------------57-----------------------------+------------ M. vinosum AF045876(60)
            |           +------------------------------------------------------------------------- M. anomalum mk067(35)
            |           +------------------------------------------------------------------------- M. holostei mk318(39)
            |           +------------------------------------------------------------------------- M. parlatorei mk277(44)
/-----------+           +------------------------------------------------------------------------- M. betonicae mk069(47)
|           |           +------------------------------------------------------------------------- M. pinguiculae AF045881(50)
|           |           \------------------------------------------------------------------------- M. onopordi mk104(61)
|           |                                                                        /------------ M. bistortarum mk080(76)
|           |                                                            /----100----+------------ M. bistortarum mk070(77)
|           +----------------------------100-----------------------------+------------------------ M. bistortarum mk071(75)
|           |                                                                        /------------ Bauerago vuyckii DB1895(70)
|           +-----------------------------------99-----------------------------------+------------ Bauerago abstrusa DB1888(71)
95          |                                                                        /------------ Sphac. poly. serrulati NO2(72)
|           +-----------------------------------90-----------------------------------+------------ Sphac. poly. persicariae AF444593(73)
|           +------------------------------------------------------------------------------------- M. tuberculiforme mk159(66)
|           +------------------------------------------------------------------------------------- M. bosniacum mk082(67)
|           +------------------------------------------------------------------------------------- Liroa emodensis DB1037(68)
|           +------------------------------------------------------------------------------------- M. pustulatum mk327(69)
|           \------------------------------------------------------------------------------------- M. nepalense mk103(74)
|                                                                                    /------------ Ustilentyloma spec. 3638(45)
\------------------------------------------------------------------------------------+------------ Ustilentyloma fluitans 1040(46)
