# Supplementary material for: Implications of molecular characters for the phylogeny of the Microbotryaceae (Basidiomycota: Urediniomycetes)
Source: BMC Evol Biol. 2006 Apr 25;6:35. doi: 10.1186/1471-2148-6-35 (PMC1526759; doi:10.1186/1471-2148-6-35)
Supplement: Additional File 1 — Alignment, Bootstrap trees, Trees. Dialign NEXUS file and concatenated MAFFT, PCMA, and POA NEXUS file. Likelihood and Parsimony bootstrap .tre and .log files and PhyML bootstrap .rtf file. Likelihood and Parsimony .tre, .log and .con files. [file 1471-2148-6-35-S1.zip › Bootstrap_Trees/Parsimony/MP_Bootstrap.rtf]

MAFFT MP Bootstrap 50% majority-rule consensus tree

                                                                                                      /--------- M. lychnidis dioicae mk115(7)
                                                                                              /--74---+--------- M. lychnidis dioicae ml326(9)
                                                                                     /---57---+----------------- M. lychnidis dioicae DB3636(8)
                                                                             /--72---+-------------------------- M. lychnidis dioicae ml215(10)
                                                                    /---57---+       \-------------------------- M. lychnidis dioicae mk147(11)
                                                                    |        \---------------------------------- M. violaceo irregulare ml50(12)
                                                                    |                                 /--------- M. silenes inflatae ml605(13)
                                                            /--83---+                         /--99---+--------- M. silenes inflatae ml620(14)
                                                            |       |                /---76---+       \--------- M. silenes inflatae mk208(15)
                                                   /---68---+       \-------92-------+        \----------------- M. silenes inflatae ml327(16)
                                                   |        |                        \-------------------------- M. violaceum s.str. ml344(17)
                                                   |        \--------------------------------------------------- M. major mk279(18)
                                                   |                                                  /--------- M. dianthorum ml354(25)
                                                   |                                          /--51---+--------- M. dianthorum ml328(26)
                                                   |                                          |       \--------- M. dianthorum mk167(27)
                                                   |                                 /---99---+       /--------- M. dianthorum mk119(24)
                                                   |                                 |        +--69---+--------- M. dianthorum ml622(28)
                                                   |                         /--74---+        \----------------- M. dianthorum ml333(29)
                                                   |                         |       |                /--------- M. dianthorum DB3637(30)
                                                   +-----------77------------+       \------100-------+--------- M. dianthorum ml329(31)
                                                   |                         \---------------------------------- M. dianthorum mk145(32)
                                           /--100--+                                                  /--------- M. stellariae ml352(19)
                                           |       |                                                  +--------- M. stellariae DB3633(20)
                                           |       +-----------------------100------------------------+--------- M. stellariae ml507(21)
                                           |       |                                                  +--------- M. stellariae ml48(22)
                                           |       |                                                  \--------- M. stellariae HML791(23)
                                           |       |                                                  /--------- M. saponariae mk142(33)
                                           |       |                                                  +--------- M. saponariae mk146(34)
                                           |       +------------------------97------------------------+--------- M. saponariae ml310(35)
                                  /---79---+       |                                                  \--------- M. saponariae ml373(36)
                                  |        |       |                                                  /--------- M. violaceum s.l. mk113(4)
                                  |        |       |                                          /--98---+--------- M. violaceum s.l. mk143(5)
                                  |        |       \--------------------64--------------------+----------------- M. violaceo verrucosum ml49(6)
                          /--57---+        |                                                          /--------- M. violaceum s.l. AY014238(37)
                          |       |        |                                                  /--65---+--------- M. violaceum s.l. AY014239(38)
                          |       |        |                                         /---57---+----------------- M. violaceum s.l. AY014235(39)
                          |       |        \-------------------100-------------------+-------------------------- M. violaceum s.l. AY014236(40)
                          |       \----------------------------------------------------------------------------- M. anomalum mk067(43)
                          |                                                                           /--------- M. cordae mk315(51)
                          |                                                                   /--55---+--------- M. tenuisporum mk153(53)
                          |                                                          /---97---+----------------- M. cordae AF045878(52)
                          +----------------------------54----------------------------+                /--------- M. reticulatum mk122(54)
                          |                                                          \-------99-------+--------- M. reticulatum mk123(55)
                          |                                                                           \--------- M. reticulatum mk112(56)
                          |                                                                           /--------- M. intermedium mk087(47)
                          |                                                                   /--98---+--------- M. intermedium mk088(48)
                          |                                                          /---56---+----------------- M. pinguiculae AF045881(50)
                          +----------------------------88----------------------------+-------------------------- M. betonicae mk069(49)
                 /---68---+                                                                           /--------- M. tragopo. pratensis mk158(58)
                 |        |                                                                   /--72---+--------- M. tragopo. pratensis mk148(59)
                 |        |                                                          /--100---+----------------- M. scorzonerae mk129(57)
                 |        +----------------------------97----------------------------+-------------------------- M. scorzonerae mk131(60)
                 |        |                                                                           /--------- M. shastense mk133(66)
                 |        |                                                                   /--99---+--------- M. vinosum AF045876(67)
                 |        +--------------------------------56---------------------------------+----------------- M. parlatorei mk277(63)
                 |        |                                                                           /--------- M. scabiosae NO3(44)
         /--56---+        +------------------------------------100------------------------------------+--------- M. scabiosae AF045880(45)
         |       |        |                                                                           /--------- M. stygium mk139(64)
         |       |        +------------------------------------100------------------------------------+--------- M. stygium mk140(65)
         |       |        +------------------------------------------------------------------------------------- M. holostei mk318(46)
         |       |        +------------------------------------------------------------------------------------- M. scolymi NO1(61)
         |       |        +------------------------------------------------------------------------------------- M. onopordi mk104(62)
         |       |        \------------------------------------------------------------------------------------- M. kuehneanum AF045879(68)
         |       |                                                                                    /--------- Bauerago abstrusa DB1888(41)
/--------+       \----------------------------------------100-----------------------------------------+--------- Bauerago vuyckii DB1895(42)
|        |                                                                                            /--------- M. bistortarum mk070(1)
|        |                                                                                    /--77---+--------- M. bistortarum mk080(3)
|        |                                                                           /--100---+----------------- M. bistortarum mk071(2)
|        |                                                                           |                /--------- M. bosniacum mk082(69)
|        |                                                                           +-------53-------+--------- M. pustulatum mk327(70)
100      |                                                                           |                /--------- Sphac. poly. persicariae AF444593(72)
|        \------------------------------------57-------------------------------------+-------99-------+--------- Sphac. poly. serrulati NO2(73)
|                                                                                    +-------------------------- M. nepalense mk103(71)
|                                                                                    +-------------------------- Liroa emodensis DB1037(74)
|                                                                                    \-------------------------- M. tuberculiforme mk159(75)
|                                                                                                     /--------- Ustilentyloma fluitans 1040(76)
\-----------------------------------------------------------------------------------------------------+--------- Ustilentyloma spec. 3638(77)

PCMA MP Bootstrap 50% Majority-rule consensus of 67370 trees (using tree weights)

                                                                                             /---------- M. bistortarum mk070(1)
                                                                                  /----68----+---------- M. bistortarum mk080(3)
                                                   /-------------100--------------+--------------------- M. bistortarum mk071(2)
                                                   |                                         /---------- M. bosniacum mk082(69)
                                                   |                    /---------60---------+---------- M. pustulatum mk327(70)
          /-------------------65-------------------+                    |                    \---------- M. tuberculiforme mk159(75)
          |                                        |          /---59----+                    /---------- Sphac. poly. persicariae AF444593(72)
          |                                        |          |         |         /---100----+---------- Sphac. poly. serrulati NO2(73)
          |                                        \----88----+         \---56----+--------------------- Liroa emodensis DB1037(74)
          |                                                   \----------------------------------------- M. nepalense mk103(71)
          |                                                                                  /---------- M. violaceum s.l. mk113(4)
          |                                                                       /----96----+---------- M. violaceum s.l. mk143(5)
          |                                        /--------------82--------------+--------------------- M. violaceo verrucosum ml49(6)
          |                                        |                                         /---------- M. lychnidis dioicae mk115(7)
          |                                        |          /--------------76--------------+---------- M. lychnidis dioicae ml326(9)
          |                                        |          +----------------------------------------- M. lychnidis dioicae DB3636(8)
          |                                        |          +----------------------------------------- M. lychnidis dioicae ml215(10)
          |                                        |          +----------------------------------------- M. lychnidis dioicae mk147(11)
          |                                        +----52----+----------------------------------------- M. violaceo irregulare ml50(12)
          |                                        |          |                              /---------- M. silenes inflatae ml605(13)
          |                                        |          |                   /----99----+---------- M. silenes inflatae ml620(14)
          |                                        |          |         /---77----+          \---------- M. silenes inflatae mk208(15)
          |                                        |          \---93----+         \--------------------- M. silenes inflatae ml327(16)
          |                                        |                    \------------------------------- M. violaceum s.str. ml344(17)
          |                              /---100---+---------------------------------------------------- M. major mk279(18)
          |                              |         |                                         /---------- M. stellariae ml352(19)
          |                              |         |                                         +---------- M. stellariae DB3633(20)
          |                              |         |                              /---100----+---------- M. stellariae ml507(21)
          |                              |         |                              |          +---------- M. stellariae ml48(22)
          |                              |         |                              |          \---------- M. stellariae HML791(23)
          |                              |         |          /--------53---------+          /---------- M. saponariae mk142(33)
          |                              |         |          |                   |          +---------- M. saponariae mk146(34)
          |                              |         |          |                   \----99----+---------- M. saponariae ml310(35)
          |                              |         |          |                              \---------- M. saponariae ml373(36)
          |                              |         |          |                              /---------- M. dianthorum mk119(24)
          |          /--------84---------+         \----56----+                   /----75----+---------- M. dianthorum ml622(28)
          |          |                   |                    |                   |          /---------- M. dianthorum ml354(25)
          |          |                   |                    |         /---98----+          +---------- M. dianthorum ml328(26)
          |          |                   |                    |         |         \----50----+---------- M. dianthorum mk167(27)
          |          |                   |                    |         |                    \---------- M. dianthorum ml333(29)
/---------+          |                   |                    \---92----+                    /---------- M. dianthorum DB3637(30)
|         |          |                   |                              |         /---100----+---------- M. dianthorum ml329(31)
|         |          |                   |                              \---53----+--------------------- M. dianthorum mk145(32)
|         |          |                   |                                                   /---------- M. violaceum s.l. AY014238(37)
|         |          |                   |                                        /----64----+---------- M. violaceum s.l. AY014236(40)
|         |          |                   |                              /---73----+--------------------- M. violaceum s.l. AY014239(38)
|         |          |                   \-------------100--------------+------------------------------- M. violaceum s.l. AY014235(39)
|         |          +---------------------------------------------------------------------------------- M. anomalum mk067(43)
|         |          |                                                                       /---------- M. scabiosae NO3(44)
|         |          |                                                            /---100----+---------- M. scabiosae AF045880(45)
|         |          +-----------------------------55-----------------------------+--------------------- M. holostei mk318(46)
|         |          |                                                                       /---------- M. intermedium mk087(47)
|         |          |                                                            /----97----+---------- M. intermedium mk088(48)
|         |          |                                                  /---60----+--------------------- M. betonicae mk069(49)
|         +----59----+         /-------------------92-------------------+------------------------------- M. pinguiculae AF045881(50)
|         |          |         |                                                             /---------- M. cordae mk315(51)
|         |          |         |                                                  /----67----+---------- M. cordae AF045878(52)
|         |          |         |                                        /---90----+--------------------- M. tenuisporum mk153(53)
100       |          +---68----+         /--------------73--------------+                    /---------- M. reticulatum mk122(54)
|         |          |         |         |                              \--------100---------+---------- M. reticulatum mk123(55)
|         |          |         |         |                                                   \---------- M. reticulatum mk112(56)
|         |          |         |         |                                        /--------------------- M. scorzonerae mk129(57)
|         |          |         \---67----+                              /---99----+          /---------- M. tragopo. pratensis mk158(58)
|         |          |                   |                    /---64----+         \----59----+---------- M. tragopo. pratensis mk148(59)
|         |          |                   |         /----57----+         \------------------------------- M. scorzonerae mk131(60)
|         |          |                   \---97----+          \----------------------------------------- M. scolymi NO1(61)
|         |          |                             \---------------------------------------------------- M. onopordi mk104(62)
|         |          +---------------------------------------------------------------------------------- M. parlatorei mk277(63)
|         |          |                                                                       /---------- M. stygium mk139(64)
|         |          +----------------------------------100----------------------------------+---------- M. stygium mk140(65)
|         |          |                                                                       /---------- M. shastense mk133(66)
|         |          |                                                            /----69----+---------- M. vinosum AF045876(67)
|         |          \-----------------------------85-----------------------------+--------------------- M. kuehneanum AF045879(68)
|         |                                                                                  /---------- Bauerago abstrusa DB1888(41)
|         \---------------------------------------100----------------------------------------+---------- Bauerago vuyckii DB1895(42)
|                                                                                            /---------- Ustilentyloma fluitans 1040(76)
\--------------------------------------------------------------------------------------------+---------- Ustilentyloma spec. 3638(77)
POA MP Bootstrap 50% majority-rule consensus tree

                                                                                   /-------- M. silenes inflatae ml605(13)
                                                                            /-100--+-------- M. silenes inflatae ml620(14)
                                                                    /--89---+      \-------- M. silenes inflatae mk208(15)
                                                             /--90--+       \--------------- M. silenes inflatae ml327(16)
                                                             |      \----------------------- M. violaceum s.str. ml344(17)
                                                             |                     /-------- M. lychnidis dioicae mk115(7)
                                                             |              /--78--+-------- M. lychnidis dioicae ml326(9)
                                                     /--88---+------61------+--------------- M. lychnidis dioicae ml215(10)
                                                     |       |              \--------------- M. lychnidis dioicae mk147(11)
                                             /--65---+       +------------------------------ M. lychnidis dioicae DB3636(8)
                                             |       |       \------------------------------ M. violaceo irregulare ml50(12)
                                             |       \-------------------------------------- M. major mk279(18)
                                             |                                     /-------- M. dianthorum ml354(25)
                                             |                              /--68--+-------- M. dianthorum mk167(27)
                                             |                      /--67---+--------------- M. dianthorum ml328(26)
                                             |                      |              /-------- M. dianthorum mk119(24)
                                             |               /--99--+------74------+-------- M. dianthorum ml622(28)
                                             |               |      \----------------------- M. dianthorum ml333(29)
                                             +------74-------+                     /-------- M. dianthorum DB3637(30)
                                             |               |              /-100--+-------- M. dianthorum ml329(31)
                                             |               \------75------+--------------- M. dianthorum mk145(32)
                                      /--99--+                                     /-------- M. stellariae ml352(19)
                                      |      |                                     +-------- M. stellariae DB3633(20)
                                      |      +-----------------99------------------+-------- M. stellariae ml507(21)
                                      |      |                                     +-------- M. stellariae ml48(22)
                                      |      |                                     \-------- M. stellariae HML791(23)
                                      |      |                                     /-------- M. saponariae mk142(33)
                                      |      |                                     +-------- M. saponariae mk146(34)
                              /--90---+      +-----------------100-----------------+-------- M. saponariae ml310(35)
                              |       |      |                                     \-------- M. saponariae ml373(36)
                              |       |      |                                     /-------- M. violaceum s.l. mk113(4)
                              |       |      |                              /--97--+-------- M. violaceum s.l. mk143(5)
                              |       |      \--------------62--------------+--------------- M. violaceo verrucosum ml49(6)
                              |       |                                            /-------- M. violaceum s.l. AY014238(37)
                       /--59--+       |                                            +-------- M. violaceum s.l. AY014239(38)
                       |      |       \--------------------100---------------------+-------- M. violaceum s.l. AY014235(39)
                       |      |                                                    \-------- M. violaceum s.l. AY014236(40)
                       |      |                                                    /-------- M. scabiosae NO3(44)
                       |      |                                             /-100--+-------- M. scabiosae AF045880(45)
                       |      \---------------------56----------------------+--------------- M. holostei mk318(46)
                       |                                                           /-------- M. cordae mk315(51)
                       |                                                    /--51--+-------- M. tenuisporum mk153(53)
                       |                                            /--94---+--------------- M. cordae AF045878(52)
                       |                                     /--63--+              /-------- M. reticulatum mk122(54)
                       |                                     |      \------99------+-------- M. reticulatum mk123(55)
                       |                                     |                     \-------- M. reticulatum mk112(56)
                       |                                     |                     /-------- M. intermedium mk087(47)
                       |                                     |              /--99--+-------- M. intermedium mk088(48)
               /--73---+                                     |      /--84---+--------------- M. betonicae mk069(49)
               |       +-----------------51------------------+--99--+----------------------- M. pinguiculae AF045881(50)
               |       |                                     |                     /-------- M. tragopo. pratensis mk158(58)
               |       |                                     |              /--75--+-------- M. tragopo. pratensis mk148(59)
               |       |                                     |      /--100--+--------------- M. scorzonerae mk129(57)
               |       |                                     +--88--+----------------------- M. scorzonerae mk131(60)
               |       |                                     +------------------------------ M. scolymi NO1(61)
               |       |                                     \------------------------------ M. onopordi mk104(62)
               |       |                                                           /-------- M. stygium mk139(64)
               |       +----------------------------100----------------------------+-------- M. stygium mk140(65)
               |       |                                                           /-------- M. shastense mk133(66)
        /--79--+       +----------------------------100----------------------------+-------- M. vinosum AF045876(67)
        |      |       +-------------------------------------------------------------------- M. anomalum mk067(43)
        |      |       +-------------------------------------------------------------------- M. parlatorei mk277(63)
        |      |       \-------------------------------------------------------------------- M. kuehneanum AF045879(68)
        |      |                                                                   /-------- Sphac. poly. persicariae AF444593(72)
        |      |                                                            /--99--+-------- Sphac. poly. serrulati NO2(73)
        |      |                                                    /--57---+--------------- Liroa emodensis DB1037(74)
        |      |                                                    |              /-------- M. bosniacum mk082(69)
/-------+      |                                             /--70--+------85------+-------- M. pustulatum mk327(70)
|       |      |                                             |      +----------------------- M. nepalense mk103(71)
|       |      \---------------------91----------------------+      \----------------------- M. tuberculiforme mk159(75)
|       |                                                    |                     /-------- M. bistortarum mk070(1)
|       |                                                    \---------100---------+-------- M. bistortarum mk071(2)
98      |                                                                          \-------- M. bistortarum mk080(3)
|       |                                                                          /-------- Bauerago abstrusa DB1888(41)
|       \-----------------------------------100------------------------------------+-------- Bauerago vuyckii DB1895(42)
|                                                                                  /-------- Ustilentyloma fluitans 1040(76)
\----------------------------------------------------------------------------------+-------- Ustilentyloma spec. 3638(77)

DIALIGN EXCL 0+1 MP 50% Majority-rule consensus of 45290 trees (using tree weights)

                                                                                      /----------- M. dianthorum mk119(24)
                                                                           /----65----+----------- M. dianthorum ml622(28)
                                                                           +---------------------- M. dianthorum ml354(25)
                                                                 /---95----+---------------------- M. dianthorum ml328(26)
                                                                 |         +---------------------- M. dianthorum mk167(27)
                                                                 |         \---------------------- M. dianthorum ml333(29)
                                                      /----54----+                    /----------- M. dianthorum DB3637(30)
                                                      |          |         /----98----+----------- M. dianthorum ml329(31)
                                                      |          \---62----+---------------------- M. dianthorum mk145(32)
                                                      |                               /----------- M. silenes inflatae ml605(13)
                                                      |                    /----88----+----------- M. silenes inflatae ml620(14)
                                                      |                    |          \----------- M. silenes inflatae mk208(15)
                                                      +---------59---------+---------------------- M. silenes inflatae ml327(16)
                                                      |                    \---------------------- M. violaceum s.str. ml344(17)
                                                      |                               /----------- M. stellariae ml352(19)
                                                      |                               +----------- M. stellariae DB3633(20)
                                                      +--------------85---------------+----------- M. stellariae ml507(21)
                                                      |                               +----------- M. stellariae ml48(22)
                                                      |                               \----------- M. stellariae HML791(23)
                                           /----81----+                               /----------- M. saponariae mk142(33)
                                           |          |                               +----------- M. saponariae mk146(34)
                                           |          +--------------70---------------+----------- M. saponariae ml310(35)
                                           |          |                               \----------- M. saponariae ml373(36)
                                           |          |                               /----------- M. violaceum s.l. mk113(4)
                                           |          +--------------88---------------+----------- M. violaceum s.l. mk143(5)
                                           |          +------------------------------------------- M. violaceo verrucosum ml49(6)
                                           |          +------------------------------------------- M. lychnidis dioicae mk115(7)
                                /----52----+          +------------------------------------------- M. lychnidis dioicae DB3636(8)
                                |          |          +------------------------------------------- M. lychnidis dioicae ml326(9)
                                |          |          +------------------------------------------- M. lychnidis dioicae ml215(10)
                                |          |          +------------------------------------------- M. lychnidis dioicae mk147(11)
                                |          |          +------------------------------------------- M. violaceo irregulare ml50(12)
                                |          |          \------------------------------------------- M. major mk279(18)
                                |          |                                          /----------- M. violaceum s.l. AY014238(37)
                                |          |                                          +----------- M. violaceum s.l. AY014239(38)
                                |          \-------------------100--------------------+----------- M. violaceum s.l. AY014235(39)
                                |                                                     \----------- M. violaceum s.l. AY014236(40)
                                |                                                     /----------- M. scorzonerae mk129(57)
                                |                                          /----97----+----------- M. tragopo. pratensis mk158(58)
                                +--------------------75--------------------+          \----------- M. tragopo. pratensis mk148(59)
                                |                                          \---------------------- M. scorzonerae mk131(60)
                                |                                                     /----------- M. cordae mk315(51)
                                |                                          /----69----+----------- M. tenuisporum mk153(53)
                                +--------------------93--------------------+---------------------- M. cordae AF045878(52)
                                |                                                     /----------- M. reticulatum mk122(54)
                      /---71----+-------------------------91--------------------------+----------- M. reticulatum mk123(55)
                      |         |                                                     \----------- M. reticulatum mk112(56)
                      |         |                                                     /----------- M. scabiosae NO3(44)
                      |         +-------------------------100-------------------------+----------- M. scabiosae AF045880(45)
                      |         |                                                     /----------- M. intermedium mk087(47)
                      |         +-------------------------97--------------------------+----------- M. intermedium mk088(48)
                      |         |                                                     /----------- M. stygium mk139(64)
                      |         +-------------------------98--------------------------+----------- M. stygium mk140(65)
                      |         +----------------------------------------------------------------- M. anomalum mk067(43)
                      |         +----------------------------------------------------------------- M. holostei mk318(46)
                      |         +----------------------------------------------------------------- M. betonicae mk069(49)
                      |         +----------------------------------------------------------------- M. pinguiculae AF045881(50)
                      |         +----------------------------------------------------------------- M. scolymi NO1(61)
           /----61----+         +----------------------------------------------------------------- M. onopordi mk104(62)
           |          |         +----------------------------------------------------------------- M. parlatorei mk277(63)
           |          |         +----------------------------------------------------------------- M. shastense mk133(66)
           |          |         +----------------------------------------------------------------- M. vinosum AF045876(67)
           |          |         \----------------------------------------------------------------- M. kuehneanum AF045879(68)
           |          |                                                               /----------- Bauerago abstrusa DB1888(41)
           |          +------------------------------99-------------------------------+----------- Bauerago vuyckii DB1895(42)
           |          |                                                               /----------- Sphac. poly. persicariae AF444593(72)
/----------+          +------------------------------96-------------------------------+----------- Sphac. poly. serrulati NO2(73)
|          |          +--------------------------------------------------------------------------- M. bosniacum mk082(69)
|          |          +--------------------------------------------------------------------------- M. pustulatum mk327(70)
|          |          +--------------------------------------------------------------------------- M. nepalense mk103(71)
|          |          +--------------------------------------------------------------------------- Liroa emodensis DB1037(74)
96         |          \--------------------------------------------------------------------------- M. tuberculiforme mk159(75)
|          |                                                                          /----------- M. bistortarum mk070(1)
|          \-----------------------------------100------------------------------------+----------- M. bistortarum mk071(2)
|                                                                                     \----------- M. bistortarum mk080(3)
|                                                                                     /----------- Ustilentyloma fluitans 1040(76)
\-------------------------------------------------------------------------------------+----------- Ustilentyloma spec. 3638(77)
